# Engineering *p*‐Orbital States via Molecular Modules in All‐Organic Electrocatalysts toward Direct Water Oxidation

**DOI:** 10.1002/advs.202410507

**Published:** 2024-12-11

**Authors:** Li‐Hong Yu, Xue‐Feng Zhang, Zi‐Ming Ye, Hong‐Gang Du, Li‐Dong Wang, Ping‐Ping Xu, Yuhai Dou, Li‐Ming Cao, Chun‐Ting He

**Affiliations:** ^1^ Key Lab of Fluorine and Silicon for Energy Materials and Chemistry of Ministry of Education College of Chemistry and Materials Jiangxi Normal University Nanchang 330022 China; ^2^ Department of Chemistry Northwestern University Evanston IL 60208 USA; ^3^ Institute of Energy Materials Science University of Shanghai for Science and Technology Shanghai 200093 China

**Keywords:** catalytic mechanism, covalent organic frameworks (COFs), electrocatalysts, oxygen evolution reaction (OER)

## Abstract

Oxygen evolution reaction (OER) is an indispensable anode reaction for sustainable hydrogen production from water electrolysis, yet overreliance on metal‐based catalysts featured with vibrant *d*‐electrons. It still has notable gap between metal‐free and metal‐based electrocatalysts, due to lacking accurate and efficient *p*‐band regulation methods on non‐metal atoms. Herein, a molecular modularization strategy is proposed for fine‐tuning the *p*‐orbital states of series metal‐free covalent organic frameworks (COFs) for realizing OER performance beyond benchmark precious metal catalysts. Optimized combination of benzodioxazole/benzodiimide‐based building blocks achieves an impressive applied potential of 1.670 ± 0.004 V versus reversible hydrogen electrode (RHE) and 1.735 ± 0.006 V versus RHE to deliver enhanced current densities of 0.5 and 1.0 A cm^−2^, respectively. Moreover, it holds a notable charge transfer amount (stands for a long service life) within operation period that outperforms all reported metal‐free electrocatalysts. Operando differential electrochemical mass spectrometry (DEMS) with isotope labeling identifies the adsorbate evolution mechanism (AEM). A variety of spectroscopic techniques and density functional theory (DFT) calculations reveal that the *p*‐band center of these catalysts can be shifted stepwise to optimize the oxygen intermediate adsorption and lower the reaction energy barrier. This work provides a novel perspective for enhancing the electrocatalytic performance of metal‐free COFs.

## Graphical abstract

The precise tailoring of *p*‐orbital states for a series of metal‐free covalent organic frameworks (COFs) using benzodioxazole/benzodiimide building blocks with different oxygen affinities to enhance the OER performances is realized. The proposed molecular modularization strategy can significantly downshift the *p*‐band center of the active carbon sites, thereby strengthening the adsorption of oxygen intermediates, and lowering the reaction energy barrier.

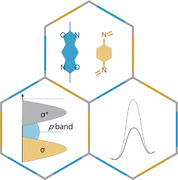

## 1 Introduction

Water electrolysis enables the production of green hydrogen employing cost‐effective electrons generated from renewable energy, contributing to carbon neutrality.^[^ ^1^ ^]^ The oxygen evolution reaction (OER) is a vital anodic reaction possessing multiple electron and proton transfer mechanisms, resulting in slow kinetics and thus limiting the efficiency of water electrolysis.^[^ ^2^ ^]^ Ir/Ru oxides featured with active and changeable *d*‐orbitals are commonly used as benchmark catalysts for OER,^[^ ^3^ ^]^ but their application is greatly hindered by low reserves and high costs. Therefore, enormous efforts have been made to manufacture alternative high‐performance catalysts using non‐precious metals or even abandoned the use of metals altogether.^[^ ^4^ ^]^ In this regard, metal‐free materials are appealing due to their high earth abundance, low cost, environmental‐friendliness, and sustainability.^[^ ^5^ ^]^ Among them, metal‐free carbon materials are representative.^[^ ^6^ ^]^ Generally, their positively charged carbon atoms are the catalytic sites for OER, yet the activity is usually unsatisfactory due to the poor variability of valence *p*‐orbitals. Defect engineering and heteroatom doping can redistribute the charge on carbon atoms, and thus are widely employed to optimize the catalytic activity of carbon catalysts.^[^ ^7^ ^]^ However, the synthesis and modification of most carbon catalysts involves pyrolysis procedures, which not only increase energy consumption, but also tend to induce unpredictable and undefined active sites. Therefore, it is imperative to develop convenient approaches to fabricate well‐defined metal‐free electrocatalysts with modulable *p*‐band structures.

**Scheme 1:**
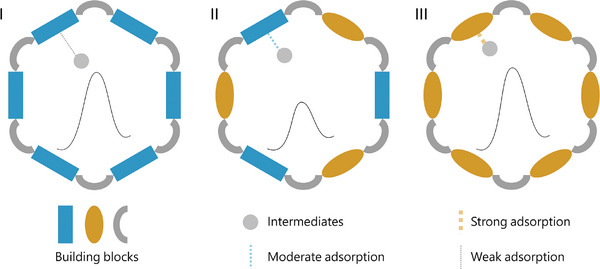
Comparative diagram of molecular modules regulating the reaction energy barriers. (I) COF assembled from building blocks containing weak oxygen affinity exhibits a higher oxygen evolution barrier; (II) COF exhibits a lower oxygen evolution barrier by precisely modulating the building blocks with strong and weak oxygen affinity; (III) COF assembled from building blocks containing strong oxygen affinity also exhibits a higher oxygen evolution barrier.

Covalent organic frameworks (COFs), a novel category of porous materials consisting of molecular building blocks (MBBs),^[^ ^8^ ^]^ are extremely promising candidates. In particular, highly conjugated and ultrathin COF nanosheets are well‐suited for electrocatalysis owing to their high specific surface areas and porosities, favorable electron transport properties, and robust framework structures.^[^ ^9^ ^]^ Moreover, COFs possess highly designable molecular structures that enable the creation of structurally modifiable and well‐defined active sites at atomic precision, facilitating the fundamental comprehension of structure‐activity relationships. At present, a few COFs have been demonstrated as OER electrocatalysts;^[^ ^10^ ^]^ nevertheless, their performance enhancement strategies are generally limited to heteroatom regulation on the backbones, which restricts further exploitation of their catalytic potential. It is a major challenge to develop novel strategies to boost the OER performance of metal‐free COF electrocatalysts. Generally, the active sites for electrocatalysis are the positively charged carbon atoms in MBBs of COFs.^[^ ^11^ ^]^ Equipping MBBs with strong/weak oxygen affinity would result in excessively strong/weak adsorption strength for oxygen intermediates thus leading to high energy barrier for OER (**Scheme** **1**). Based on the Sabatier principle, rational tailoring of MBB to achieve moderate adsorption strength for oxygen intermediates would substantially lower the energy barrier, yet such fine‐tuning is rather difficult in the same MBB. The combination of MBBs with different oxygen binding strengths will be a relatively easy way to achieve this target (Scheme 1), being similar to the bimetallic regulation in traditional inorganic metal‐based materials. However, it remains unexplored to regulate the OER activity and the corresponding enhancement mechanism by precisely modulating MBBs with opposite oxygen affinities.

In this work, we report a novel molecular modularization strategy for stepwise regulating the OER performances of series multi‐component metal‐free COFs. Electrochemical experiments revealed that the apparent activation energy of COFs could be significantly reduced by adjusting the ratio of benzodioxazole units with weak oxygen affinity to benzodiimide units with strong oxygen affinity. The optimal benzodioxazole‐benzodiimide hybrid COF demands low applied potentials of 1.541 ± 0.002 V versus reversible hydrogen electrode (RHE) at 0.01 A cm^−2^ on glassy carbon electrode (GCE), and 1.653 ± 0.011 V versus RHE and 1.757 ± 0.021 V versus RHE at 0.5 and 1.0 A cm^−2^ on carbon cloth (CC), respectively. It can achieve sustainable oxygen evolution with negligible performance degradation for 250 h at a high current density of 100 mA cm^−2^, surpassing all metal‐free electrocatalysts. Multiple spectroscopic techniques combined with density functional theory (DFT) calculations elucidated that the introduction of building blocks with strong oxygen affinity into benzodioxazole‐based COF can significantly downshift the *p*‐band center of the active carbon sites, thereby strengthening the adsorption of oxygen intermediates, and lowering the reaction energy barrier.

## 2 Results and Discussion

**Figure 1:**
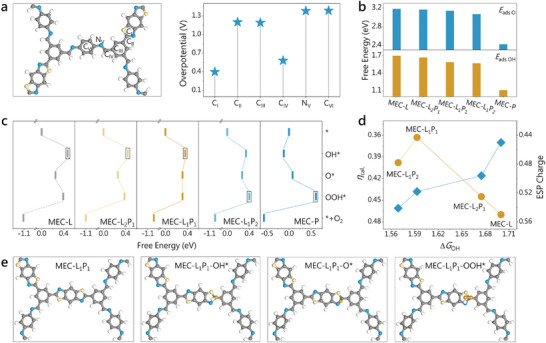
Theoretical prediction. a) Different potential catalytic sites of benzodioxazole/benzodiimide‐based COF and their corresponding theoretical overpotentials for OER. b) Adsorption energies of O and OH intermediates on the MEC‐L*_x_* P*_y_*. c) Free energy profiles of OER pathways for MEC‐L*_x_* P*_y_*. d) Plot of theoretical overpotentials and ESP charges on active sites of MEC‐L*_x_* P*_y_* with OH adsorption energies. e) Structural models of MEC‐L_1_P_1_ without and with OH, O, or OOH adsorption.

Benzodioxazole and benzodiimide are building blocks commonly adopted to fabricate highly conjugated COFs.^[^ ^12^ ^]^ DFT calculations revealed that benzodioxazole features a weak oxygen affinity while benzodiimide features a strong one. Therefore, we envisaged enhancing the OER activity of COFs though regulating these two typical building blocks with different oxygen affinities. In order to explore the optimal active sites in COFs, we calculated the Gibbs free energy change (Δ*G*) for each fundamental step in OER for six possible catalytic sites in the COFs. The carbon (I) in the O−C = N of the oxazole ring structure was found to be the best catalytic site for OER, possessing the lowest theoretical overpotential (**Figure** **1**a). Afterward, we built structural models of multiple COFs and calculated their Δ*G* to determine the optimal ratio of different building blocks. As the proportion of benzodiimide units in COFs increases, their adsorption energies for O and OH gradually decrease (Figure 1b). Particularly, MEC‐L (MEC: molecule‐enhanced catalyst) linked by benzodioxazole contains the highest density of active sites, yet the adsorption strength of its carbon active center to the oxygen intermediates is too weak, resulting in a high energy barrier for its rate‐determining step (RDS) for the OH generation (Figure 1c). The introduction of benzodiimide will boost the binding affinity of the carbon sites in benzodioxazole for oxygen species, thereby lowering the RDS energy barrier. At the identical ratio of these two building blocks, MEC‐L_1_P_1_ exhibits the optimal adsorption strength for oxygen intermediates with a reduced RDS barrier of 1.59 eV (Figure 1c), demonstrating the smallest theoretical overpotential (Figure 1d). Further elevating the proportion of benzodiimide in the COFs will not only excessively enhance the adsorption strength, leading to a greater obstacle in OOH formation (Figure 1c), but also make the density of active sites in their frameworks lower, thereby increasing the overall overpotentials for OER (Figure 1d). The model plots of the key structures visually present the binding of the oxygen intermediates to the carbon active sites (Figure 1e; Figures S1–S4, Supporting Information). Charge analysis unveiled that the positive charges of the active carbon sites in benzodioxazole gradually increase with increasing the proportion of benzodiimide units in COFs, promoting the adsorption energies of the hydroxyl intermediates (Figure 1d). However, it is evident from the volcano diagram of the variation of theoretical overpotential with OH adsorption energy that only moderate binding strength will afford an optimal overpotential. This is consistent with the Sabatier principle in traditional metal catalysts.

**Figure 2:**
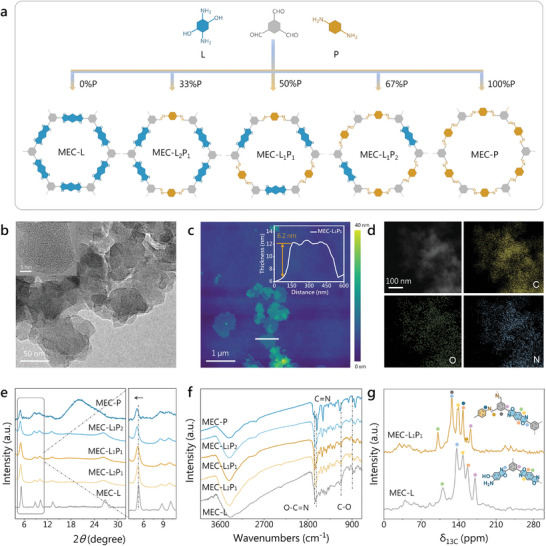
Synthesis and structural characterizations. a) Schematic representation for the synthesis of MEC‐L*_x_* P*_y_*. b) TEM image (inset: HRTEM image), c) AFM image (inset: thickness measurement), and d) EDX elemental mapping images of MEC‐L_1_P_1_. e) PXRD patterns, and f) FT‐IR spectra of MEC‐L*_x_* P*_y_*. g) Solid‐state ^13^C CP‐MAS NMR spectra of MEC‐L_1_P_1_ and MEC‐L.

MEC‐L linked by benzodioxazole was constructed via the condensation reaction of 1,3,5‐triformylbenzene with 2,5‐diamino‐1,4‐dihydroxybenzene dihydrochloride (DDB) (**Figure** **2**a). In order to incorporate different contents of benzodiimide into the frameworks, we fabricated multiple COFs with different ratios of *p*‐phenylenediamine (PPD) instead of DDB, named MEC‐L_2_P_1_, MEC‐L_1_P_1_, MEC‐L_1_P_2_ and MEC‐P, respectively (Figure S5, Supporting Information). The scanning electron microscope (SEM) and transmission electron microscope (TEM) images unveil that MEC‐L_2_P_1_, MEC‐L_1_P_1_, and MEC‐L_1_P_2_ show nanosheet morphologies with similar sizes (Figure 2b; Figure S6, Supporting Information) while MEC‐L and MEC‐P are large‐sized nanosheets (Figure S6, Supporting Information). High‐resolution TEM (HRTEM) images show the absence of lattice fringes in all the as‐prepared COFs except MEC‐L (Figure 2b; Figure S6, Supporting Information), indicating their low crystallinity. Atomic force microscopy (AFM) analysis further revealed that they all possess a sheet‐like morphology with close thickness (≈6 nm) (Figure 2c; Figures S7–S10, Supporting Information). Energy dispersive X‐ray (EDX) elemental mapping images confirm the existence of evenly distributed C, N, and O elements in MEC‐L_1_P_1_ (Figure 2d). Powder X‐ray diffraction (PXRD) pattern reveals that MEC‐L has diffraction peaks matching those of the reported LZU‐190 (Figure 2e),^[^ ^13^ ^]^ indicating the successful synthesis of benzodioxazole‐linked framework structure. With the partial substitution of building block benzodioxazole by benzodiimide, the characteristic diffraction peaks of MEC‐L were still observed in MEC‐L_2_P_1_, MEC‐L_1_P_1_, and MEC‐L_1_P_2_, demonstrating that they are all structurally isomorphic. However, MEC‐P exhibited only diffraction peaks corresponding to the (100) and (110) faces, indicating that the complete substitution of benzodioxazole by benzodiimide resulted in a decrease in its crystallinity. As the proportion of benzodiimide in COFs increased, their diffraction peaks gradually shifted to lower angles, implying enlargement of their crystal cells. In addition, the PXRD refinements of MEC‐L and MEC‐L_1_P_1_ further confirmed that they are isomorphic with LZU‐190 and the introduction of benzodiimide makes the unit cell expansion (Figure S11, Supporting Information). Fourier transform infrared (FT‐IR) spectroscopy uncovered that the peaks at 1668, 1418, and 1116 cm^−1^ of MEC‐L are assigned to the stretching vibration of O─C═N, the symmetric and asymmetric stretching vibrations of C─O─C in benzoxazole (Figure 2f), respectively, further confirming the formation of benzodioxazole structure.^[^ ^13^, ^14^ ^]^ MEC‐P presents a peak at 1620 cm^−1^ belonging to the C═N stretching vibration,^[^ ^15^ ^]^ indicating the successful synthesis of benzodiimide structure. Moreover, the infrared characteristic peaks of benzoxazole and imine can be observed in MEC‐L_2_P_1_, MEC‐L_1_P_1_, and MEC‐L_1_P_2_, signifying that they simultaneously comprised benzodioxazole and benzodiimide modules. In addition, the characteristic peaks of benzoxazole gradually weaken with the increase of PPD feeding ratio, while those of imine gradually strengthen, implying that the content of benzodiimide in COFs increases. Raman spectra revealed that MEC‐L_2_P_1_, MEC‐L_1_P_1_, and MEC‐L_1_P_2_ possess characteristic peaks originating from O─C═N in benzodioxazole and C═N in benzodiimide (Figure S12, Supporting Information). Solid‐state ^13^C cross‐polarization (CP) magic angle spinning (MAS) nuclear magnetic resonance (NMR) spectrum uncovered that MEC‐L_1_P_1_ features signal peaks at 139, 147, and 161 ppm originating from carbon atoms within the oxazole ring (Figure 2g), testifying again the successful formation of benzoxazole structure.^[^ ^13^, ^14^ ^]^ Accompanying the increase of PPD feeding, both the proportion of O 1*s* in the full X‐ray photoelectron spectroscopy (XPS) spectra and the proportion of C─O─C in the high‐resolution O 1*s* spectra decrease (Figures S13 and S14, Supporting Information), indicating that benzodioxazole is gradually replaced by benzodiimide in the series COFs. The signal peaks of C−O/C = N bonds were detected in both high‐resolution XPS spectra of C 1*s* and N 1*s* (Figure S15, Supporting Information), originating from the benzoxazole or imine structures.^[^ ^14^, ^16^ ^]^ Nitrogen (77 K) sorption measurements unveiled that MEC‐L_2_P_1_, MEC‐L_1_P_1_, and MEC‐L_1_P_2_ with microporous structures showcase approximate Brunauer−Emmett−Teller specific surface areas of 519.0, 581.6 and 554.6 m^2^ g^−1^ (Figure S16, Supporting Information), respectively, which should be favorable for electrocatalytic mass transfer. Thermogravimetric analysis unveiled that these series COFs display great thermal stability with the thermal decomposition temperature reaching up to 300 °C (Figure S17, Supporting Information), possibly due to their highly conjugated structures.

**Figure 3:**
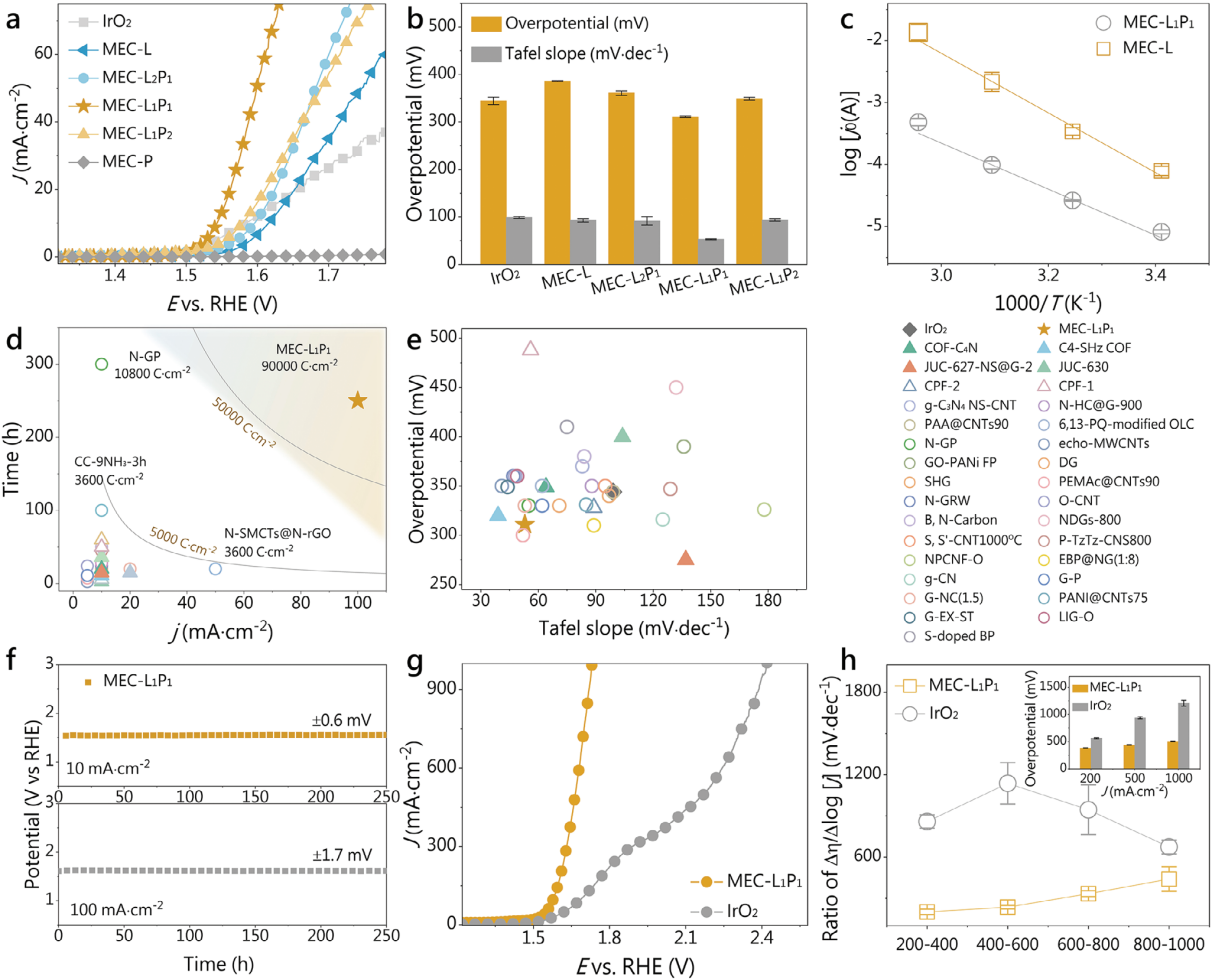
Electrocatalytic performance evaluation. a) LSV curves, b) Tafel slopes and overpotentials (*η*) at 10 mA cm^−2^ of MEC‐L*_x_* P*_y_* and IrO_2_ on GCE. c) Arrhenius plots of MEC‐L_1_P_1_ and MEC‐L. d) Comparison of the total amount of charge transferred between MEC‐L_1_P_1_ and the reported metal‐free catalyst during the test time of electrochemical stability. e) Comparison of OER performances of MEC‐L_1_P_1_ and the recently reported metal‐free catalysts. The solid triangle represents metal‐free COF, the hollow triangle represents metal‐free porous organic polymers, and the hollow circle represents metal‐free carbon‐based catalysts. Detailed data are available in Table S1 (Supporting Information). f) Constant current chronopotentiometry measurements. g) LSV curves of MEC‐L_1_P_1_ and IrO_2_ on CC. h) Ratios of Δ*η* to Δlog|*j*| at different current density intervals, inset: overpotentials at different current densities for MEC‐L_1_P_1_ and IrO_2_. Data error bars were calculated from three replicate experiments.

The electrochemical OER properties of these COFs were measured on a GCE in alkaline media. The performance of commercial IrO_2_ with the same loading was also measured as a comparison. The linear sweep voltammetry (LSV) curves with 95% *iR* compensation illustrate that MEC‐L_1_P_1_ possesses the best catalytic activity with a low overpotential of 311 ± 2 mV at 10 mA cm^−2^ (**Figure** **3**a; Figures S18 and S19, Supporting Information), outperforming the benchmark IrO_2_ (344 ± 7 mV). Notably, the experimentally measured overpotentials of COFs are in the order of MEC‐L_1_P_1_<MEC‐L_1_P_2_<MEC‐L_2_P_1_<MEC‐L (Figure 3a,b), which is in agreement with the theoretical calculations. It is worth noting that the OER performance of MEC‐L_1_P_1_ is much better than that of the mechanically mixed MEC‐L/MEC‐P (Figure S20, Supporting Information), suggesting that the coexistence of benzodioxazole and benzodiimide in the identical molecular framework can enhance intrinsic activity. LSV‐derived Tafel plots yield information about OER kinetics. MEC‐L_1_P_1_ exhibits the optimum Tafel slope of 53 ± 1 mV dec^−1^ among these catalysts, substantiating its faster OER kinetics (Figure S21, Supporting Information). Moreover, MEC‐L_1_P_1_ possesses the best turnover frequency of 10.45 ± 0.13 s^−1^ at an overpotential of 350 mV (Figure S22, Supporting Information). Arrhenius plot according to the variation of OER activity with temperature uncovered a linear correlation between the apparent activation energy (*E*_a_) of electrocatalyst and its catalytic activity (Figure 3c; Figure S23, Supporting Information). The fitted *E*_a_ MEC‐L_1_P_1_ (71.2 ± 8.2 kJ mol^−1^) is much lower than that of MEC‐L (105.7 ± 0.1 kJ mol^−1^), indicating that the incorporation of benzodiimide building block can facilitate intrinsic activity of the active sites. Notably, the OER performance of MEC‐L_1_P_1_ outperforms a majority of reported advanced metal‐free electrocatalysts (Figure 3e; Table S1, Supporting Information) and even some efficient metal‐based electrocatalysts (Figure S24 and Table S2, Supporting Information). The electrocatalytic stability is also essential for the practicability of catalysts. Constant current chronopotentiometry measurements displayed that MEC‐L_1_P_1_ can operate stably at 10 and 100 mA cm^−2^ for 250 h (Figure 3f) with potential fluctuation of merely 0.6 and 1.7 mV, respectively. To the best of our knowledge, the charge transfer amount (*Q*) calculated by multiplying the operation time by the catalytic current density outperforms all reported metal‐free electrocatalysts (Figure 3d; Figure S25 and Table S1, Supporting Information), confirming their remarkable service life. The polarization curve of MEC‐L_1_P_1_ after 10 000 cyclic voltammetry (CV) scans almost duplicated the initial one, also demonstrating its superior electrochemical stability (Figure S26, Supporting Information). Furthermore, MEC‐L_1_P_1_ provides a superior electrochemical double layer capacitance value and a higher electrochemically active specific surface area (Figures S27 and S28, Supporting Information). Electrochemical impedance profiles unveil that the impedance values of MEC‐L_1_P_1_, MEC‐L_1_P_2_, and MEC‐L_2_P_1_ are approximated and significantly smaller than those of MEC‐L and MEC‐P (Figure S29, Supporting Information), indicating that the simultaneous inclusion of benzodioxazole and benzodiimide building blocks can also enhance the charge transfer capacities of the all‐organic electrocatalysts.

The performance of MEC‐L_1_P_1_ at enhanced current densities was investigated by employing CC as the working electrode. MEC‐L_1_P_1_/CC exhibits remarkable OER activity (Figure 3g), requiring an overpotential of 384 ± 3 mV at 200 mA cm^−2^. Particularly, it yields enhanced current densities of 0.5 and 1.0 A cm^−2^ at overpotentials of 440 ± 4 and 505 ± 6 mV (Figure 3g; Figure S30, Supporting Information), respectively, both significantly outperforming IrO_2_/CC. MEC‐L_1_P_1_ presents lower ratios of Δ*η* to Δlog|*j*| in all the identical current density intervals (Figure 3h), further indicating its preferred activity over IrO_2_. The post‐OER characterizations uncovered that MEC‐L_1_P_1_ can well maintain the initial chemical composition as well as framework structure (Figures S31 and S32, Supporting Information), corroborating its favorable structural stability in electrocatalytic OER.

**Figure 4:**
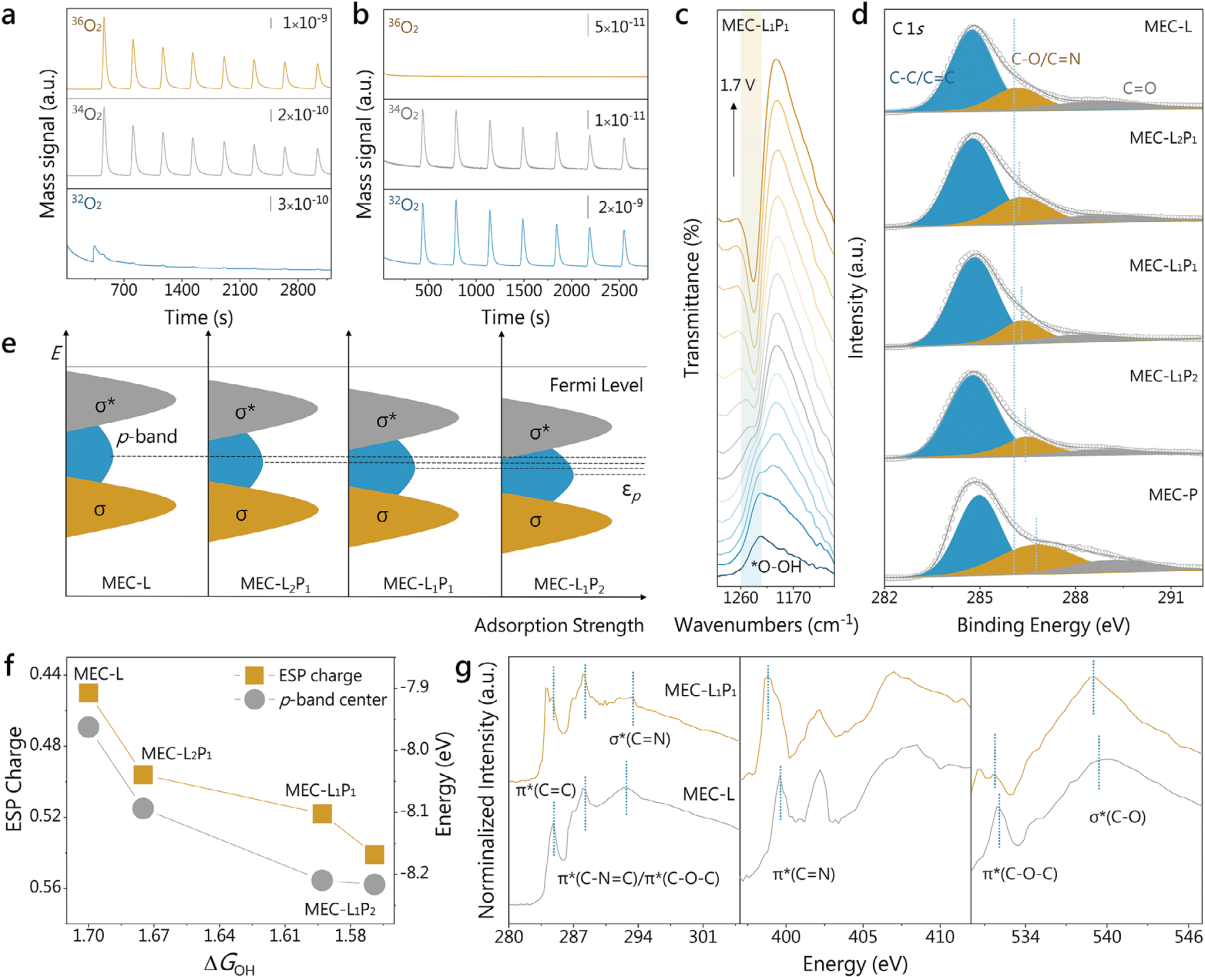
Modulation mechanism of catalytic activity. Operando DEMS with isotope labeling experiments at a) H_2_^18^O, b) H_2_^16^O of MEC‐L_1_P_1_. c) Potential‐dependent operando ATR‐SEIRAS. d) High resolution XPS spectra of C 1*s* in MEC‐L*_x_* P*_y_*. e) The *p*‐orbital state diagrams with corresponding *p*‐band centers of MEC‐L*_x_* P*_y_*. f) Δ*G*_OH_ of MEC‐L*_x_* P*_y_* varies with ESP charges or *p*‐band centers of active sites. g) NEXAFS spectra of the C *K*‐edge, N *K*‐edge, and O *K*‐edge for MEC‐L_1_P_1_ and MEC‐L, respectively.

To determine the reaction mechanism of MEC‐L_1_P_1_, the OER products of the electrochemical process were investigated using operando differential electrochemical mass spectrometry (DEMS) with isotope labeling experiments (**Figure** **4**a,b). We performed two set of DEMS experiments with H_2_^18^O and H_2_^16^O as electrolytes (1 M KOH), respectively. First, the working electrode loaded with MEC‐L_1_P_1_ was placed in H_2_^18^O electrolyte for CV measurements, and the molecular weight of the generated oxygen was monitored in real time. The results revealed that MEC‐L_1_P_1_ yielded ^34^O_2_ and ^36^O_2_ products, indicating that it followed adsorbate evolution mechanism (AEM).^[^ ^17^ ^]^ Subsequently, the catalyst was washed with a large amount of water (H_2_^16^O) and the same CV measurements were run in the H_2_^16^O electrolyte while monitoring the generated oxygen in real time. MEC‐L_1_P_1_ only produced ^32^O_2_ and ^34^O_2_, further illustrating that MEC‐L_1_P_1_ followed AEM. Additionally, potential‐dependent operando attenuated‐total‐reflection surface enhanced infrared adsorption spectroscopy (ATR‐SEIRAS) revealed that a characteristic peak attributed to the OOH intermediate can be observed at 1224 cm^−1^ (Figure 4c; Figures S33 and S34, Supporting Information), further corroborating that the electrocatalytic OER by MEC‐L_1_P_1_ followed the AEM pathway. DFT calculations were implemented to unveil the underlying reasons for the high activity of MEC‐L_1_P_1_. We analyzed the *p*‐band structures of the series COFs based on the calculated density of state of *p*‐orbital on the active sites (Figure 4e). With the elevated benzodiimide content in COFs, the *p*‐band centers of the active carbon atoms moved further away from the Fermi energy level (Figure 4e), resulting in more electron‐deficient carbon sites with increasing carried positive charges, which can significantly enhance the adsorption energy of OH intermediate (Figure 4f). However, too strong or too weak adsorption strengths are detrimental to catalysis, and MEC‐L_1_P_1_ with appropriate combination of molecular modules possesses relative moderate adsorption strength, thus resulting in optimal activity. Methanol oxidation reaction experiments revealed that MEC‐L_1_P_1_ exhibits moderate surface coverage of OH (Figure S35, Supporting Information),^[^ ^18^ ^]^ being consistent with its moderate OH adsorption strength. XPS and X‐ray absorption spectroscopy were implemented to further verify these theoretical results. High‐resolution C 1*s* XPS spectroscopic analysis revealed a gradual positive shift of the binding energy peaks with elevated benzodiimide content in COFs (Figure 4d), indicating that their carbon atoms are more electron deficient. Moreover, we measured the near‐edge X‐ray absorption fine structures (NEXAFS) of the C *K*‐edge, O *K*‐edge, and N *K*‐edge of MEC‐L_1_P_1_ and MEC‐L (Figure 4g), respectively. The NEXAFS patterns of MEC‐L and MEC‐L_1_P_1_ present the characteristic peaks of π*(C–N = C), π*(C = N), π*(C–O) and σ*(C–O), demonstrating the successful construction of the benzodioxazole structure. The major characteristic peaks in the C *K*‐edge of MEC‐L_1_P_1_ are shifted to higher energies compared with MEC‐L, signifying the loss of electrons from carbon atoms,^[^ ^19^ ^]^ being coincident with the XPS result and DFT calculations.

## 3 Conclusion

In summary, we report a unique strategy to tailor the *p*‐orbital states of metal‐free COFs by precisely modulating MBBs with different oxygen affinities, thus realizing the systematic regulation of OER performances. Electrochemical measurements and a variety of spectral characterization techniques combined with DFT theoretical calculations verified that the molecular modularization strategy fundamentally enhances intrinsic activities of the multicomponent COFs through adjusting their *p*‐band centers. Consequently, the optimized MEC‐L_1_P_1_ exhibits an impressive OER performance in alkaline condition, yielding enhanced current densities at low overpotentials and excellent electrochemical durability, being even comparable to the advanced metal‐based catalysts reported so far. Our research not only elucidates the relationship between molecular modules and water oxidation activity, but also explores the potential of all‐organic electrocatalysts for efficient and economical access to chemicals.

## Conflict of Interest

The authors declare no conflict of interest.

## Supporting information



Supporting Information

## Data Availability

The data that support the findings of this study are available from the corresponding author upon reasonable request.
