# Supplementary material for: Engineering p‐Orbital States via Molecular Modules in All‐Organic Electrocatalysts toward Direct Water Oxidation
Source: Adv Sci (Weinh). 2024 Dec 11;12(5):2410507. doi: 10.1002/advs.202410507 (PMC11792050; doi:10.1002/advs.202410507)
Supplement: Supplementary file 1 — Supporting Information [file ADVS-12-2410507-s001.pdf]

ADVANCED SCIENCE
Open Access

Supporting Information

for *Adv. Sci.*, DOI 10.1002/advs.202410507

Engineering *p*-Orbital States via Molecular Modules in All-Organic Electrocatalysts toward Direct Water Oxidation

Li-Hong Yu, Xue-Feng Zhang, Zi-Ming Ye, Hong-Gang Du, Li-Dong Wang, Ping-Ping Xu, Yuhai Dou, Li-Ming Cao and Chun-Ting He**

Supporting Information

Engineering *p*-Orbital States via Molecular Modules in All-organic Electrocatalysts toward Direct Water Oxidation

Li-Hong Yu,[a] Xue-Feng Zhang,[a] Zi-Ming Ye,[b] Hong-Gang Du,[a] Li-Dong Wang,[a] Ping-Ping Xu,[a] Yuhai Dou,[c] Li-Ming Cao,[a] and Chun-Ting He[a]**

[a] Key Lab of Fluorine and Silicon for Energy Materials and Chemistry of Ministry of Education, College of Chemistry and Materials, Jiangxi Normal University, Nanchang 330022, China

[b] Department of Chemistry, Northwestern University, Evanston, IL 60208, USA

[c] Institute of Energy Materials Science, University of Shanghai for Science and Technology, Shanghai 200093, China

Table of Contents

Experimental Section……………………………………………………………………………………3
Figure S1. Structure models of MEC-L for key intermediates of AEM reaction pathway……………………8
Figure S2. Structure models of MEC-L_2P_1 for key intermediates of AEM reaction pathway………………9
Figure S3. Structure models of MEC-L_1P_2 for key intermediates of AEM reaction pathway………………10
Figure S4. Structure models of MEC-P for key intermediates of AEM reaction pathway………………11
Figure S5. Optical photographs of catalysts……………………………………………………………12
Figure S6. Morphological characterization of catalysts…………………………………………………13
Figure S7. AFM image and thickness of MEC-L…………………………………………………………14
Figure S8. AFM image and thickness of MEC-L_2P_1……………………………………………………15
Figure S9. AFM image and thickness of MEC-L_1P_1……………………………………………………16
Figure S10. AFM image and thickness of MEC-P…………………………………………………………17
Figure S11. Observed PXRD pattern and refined modeling profile of catalysts……………………………18
Figure S12. Raman spectra of catalysts……………………………………………………………………19
Figure S13. XPS survey scans of catalysts…………………………………………………………………20
Figure S14. High-resolution O 1*s* XPS spectra of catalysts………………………………………………21
Figure S15. High-resolution N 1*s* XPS spectra of catalysts………………………………………………22
Figure S16. N_2 sorption curves and pore size distribution of catalysts…………………………………23
Figure S17. TG curves and DTG curves of catalysts………………………………………………………24
Figure S18. LSV curves of catalysts………………………………………………………………………25
Figure S19. LSV curves of MEC-L_1P_1 with 95% and 0% *iR* compensation……………………………26
Figure S20. LSV curves of MEC-L_1P_1 and MEC-L/MEC-P………………………………………………27
Figure S21. Tafel slopes of catalysts………………………………………………………………………28
Figure S22. Redox area curve and TOF value of catalysts…………………………………………………29
Figure S23. LSV curves of catalysts tested at different temperatures………………………………………30
Figure S24. Comparison of electrocatalytic performance of catalysts………………………………………31
Figure S25. Comparison of electrocatalytic performance of catalysts (COF) ………………………………32
Figure S26. LSV curves of MEC-L_1P_1 before and after 10000 cycles of CV scan…………………………33
Figure S27. CV curves of catalysts on GCE and C_{dl} comparison of catalysts…………………………………34
Figure S28. ECSA value of catalysts………………………………………………………………………35
Figure S29. Electrochemical impedance profiles of MEC-L_xP_y…………………………………………36
Figure S30. LSV curves of catalysts supported on carbon cloth……………………………………………37
Figure S31. PXRD patterns of catalysts before and after electrocatalytic test………………………………38
Figure S32. FTIR spectra of catalysts before and after electrocatalytic test…………………………………39
Figure S33. Potential-dependent operando ATR-SEIRAS of MEC-L_1P_1…………………………………40
Figure S34. Operando ATR-SEIRAS system configuration…………………………………………………41
Figure S35. The LSV curves of catalysts…………………………………………………………………42
Table S1. Comparison of OER performance of MEC-L_1P_1 with metal-free electrocatalysts………………43
Table S2. Comparison of OER performance of MEC-L_1P_1 with metal-based electrocatalysts………………46
References……………………………………………………………………………………………47

Experimental Section

Materials

2,5-diamino-1,4-benzenediol dihydrochloride (97%), 1,3,5-triformylbenzene (96%) were acquired from *Adamas*. *N*,*N*-dimethylformamide (DMF, 99%), tetrahydrofuran (THF, AR, 99%) and *p*-phenylenediamine (PPD, 97%) were purchased from *Aladdin*. Benzimidazole (96%), Nafion 117 solution (~5% in a mixture of lower aliphatic alcohols and water), potassium hydroxide (KOH, semiconductor grade, 99.99%), mesitylene (98%, extra dry, with molecular sieves, water ≤ 50 ppm (by K.F.), Energyseal), and *N*-methyl pyrrolidone (NMP, 99%) were purchased from *Energy Chemical*. Carbon cloth (CC, WOS1009) was obtained from *Phychemi* company. The conductive substrates described above were cut into the size as required, washed several times with distilled water, ethanol and acetone, respectively, then boiled in HNO_3 solution (6 M) at 90°C for 1 h, and finally washed with deionized water to neutrality, and dried in vacuum oven at 60°C for 12 h. Copper foam (CF) was gained from *Jiangteng Electronics* Co., LTD. The substrate was first ultrasonic with acetone and ethanol, then immersed in HCl solution (3 M), then washed and neutralized with ultra-pure water, dried in vacuum oven. Ultrapure water (resistivity ≥ 18 MΩ·cm, conductivity ≤ 0.1 μs·cm^{-1}, TOC ≤ 10 ppb) was obtained from ultrapure water preparation machine (Dingji, Milli-Q, IQ7003).

Synthesis of MEC-L$_x$P$_y$:

The synthesis method of LZU-190 was based on the reported literature[1]. 2,5-diamino-1,4-benzenediol dihydrochloride, *p*-phenylenediamine, 1,3,5-triformylbenzene (0.054 g, 0.335 mmol), and benzimidazole (0.177 g, 1.50 mmol) were transferred to a Pyrex tube. After that, reaction solvents such as NMP and mesitylene (*V*/*V* = 1/1, 2.25 mL: 2.25 mL) were added to the above solid powders. The mixed solution was dispersed homogeneously by sonication and rapidly frozen in a 77 K liquid nitrogen bath. Subsequently, the system was evacuated to remove oxygen and other gaseous impurities to provide anaerobic reaction conditions. Upon warming

to room temperature, set the reaction at 185°C and left undisturbed for 5 days. After the reaction was completed, the solid substance was centrifuged, washed with acetone and THF, and dried at room temperature. And then, DMF was used as solvent to extract the residual monomers or oligomers by Soxhlet extraction for 2 days. At last, the powder was washed with methanol, and freeze-dried to obtain the products. In this experiment, the ratio of benzodioxazole and benzodiimide in covalent organic frameworks (COFs) was regulated by precisely adjusting the amount of 2,5-diamino-1,4-benzenediol dihydrochloride and *p*-phenylenediamine in the reaction system. To prepare the series of MEC-L_xP_y, the molar ratios of 2,5-diamino-1,4-benzenediol dihydrochloride and *p*-phenylenediamine were set as 1:0 (MEC-L), 2:1 (MEC-L_2P_1), 1:1 (MEC-L_1P_1), 1:2 (MEC-L_1P_2), 0:1 (MEC-P), respectively.

Electrochemical Measurements Methods

All the electrochemical performances were evaluated on the electrochemical workstation (CHI760E A19012b) using the classic three-electrode system in 1.0 M KOH electrolyte at room temperature. The electrolyte was saturated with oxygen for 1 h before the measurements. All the electrochemical tests were used saturated Hg/HgO electrode as the reference electrode, platinum plate as counter electrode, and glassy carbon electrode (GCE) or carbon cloth (CC) coated with catalysts as working electrodes. The area of glass carbon electrode is 0.2 cm^2. The size of carbon cloth as the working electrode is 0.5 cm × 0.2 cm. To generate the catalyst ink, 3 mg catalyst, 100 μL of H_2O, 500 μL of isopropanol and 50 μL of 5 wt% Nafion solution were mixed and sonicated. 10 μL catalyst ink was dropped on GCE and then dried at room temperature for 6 h to afford the catalyst film. The samples were activated by cyclic voltammetry (CV) scanning to achieve a relatively stable state. Linear sweep voltammetry (LSV) was carried out at a scanning rate of 5 $mV\cdot s^{-1}$ with *iR* calibration. The CV curves of the samples measured in 1.0 M KOH solution at different scan rates (20, 40, 60, 80, 100, and 120 $mV\cdot s^{-1}$) were used to compare the electrochemically active specific surface area (ECSA). The

stability test was tested by the chronopotentiometry. The electrochemical impedance spectroscopy (EIS) was obtained in the frequency range from 100 kHz to 0.1 Hz. In addition, 30 μL of catalyst ink was dropped on different conductive supports as self-supporting working electrode.

The Tafel slope was calculated as follows:

$$\eta = a + b \log i \quad (1)$$

Where η is the overpotential, a is the Tafel constant, b is the Tafel slope, and i is the current.

The potential was calibrated against to the reversible hydrogen electrode (RHE).

$$E_{\mathrm{RHE}} = E + E_{\mathrm{Hg/HgO}} + 0.0591 \times \mathrm{pH} \quad (2)$$

The ECSA of catalysts was estimated by the electrochemical double layer capacitance.

$$\mathrm{ECSA} = \frac{C_{\mathrm{dl}}}{C_{\mathrm{s}}} \quad (3)$$

The value of C_s (specific capacitance) is 0.04 mF·cm^{-2}.

At different temperatures such as 20, 35, 50 and 65°C, the LSV curve of the catalyst on GCE was obtained by electrochemical testing and the Tafel slope of the catalyst was obtained by fitting the linear part of the Tafel plot. The apparent activation energy of OER is determined by the Arrhenius equation[2].

$$\frac{\mathrm{d}(\log j_0)}{\mathrm{d}(1/T)} = -\frac{E_{\mathrm{a,app}}}{2.303R} \quad (4)$$

Where j_0 is the exchange current density.

The TOF of catalysts was calculated according to the following formula[3]:

$$\mathrm{TOF} = \frac{J \times N_{\mathrm{A}}}{n \times F \times \tau} \quad (5)$$

Where J is the current density value under a specific overpotential value, N_A is Avogadro number, the value is 6.022×10^{23}, n is the electron transfer number in the OER reaction, the value is $n = 4$, F is the Faraday constant, the value is 96 485 C·mol^{-1}, and τ is the number of active sites involved in the electrochemical reaction. By measuring the surface area value of the

CV curve at a specific scanning rate obtained from the non-Faraday region, it is calculated using the following formula:

$$\tau = \frac{\text{area of the } C_{\text{dl}} \text{ curve}}{\text{scan rate} \times 1.601 \times 10^{-19}} \tag{6}$$

Characterization

Powder X-ray diffraction (PXRD) patterns were collected on Rigaku MiniFlex600 X-ray diffractometer (PXRD, 45 kV, 15 mA, CuKα radiation). The scanning electron microscope (SEM) and transmission electron microscope (TEM) images were characterized by Zeiss EVO 10 (*Carl Zeiss*) and JEM2100 (200 KV). Energy dispersive X-ray (EDX) elemental mapping images were acquired on the Zeiss SmartEDX equipped in Zeiss EVO 10. X-ray photoelectron spectroscopy (XPS) data were collected on the Escalab 250Xi X-ray photo-electron spectrometer. Thermogravimetric (TG) analysis was performed on TA Q50 system under a flow of N_2 by heating from room temperature to 600°C at a rate of 10°C min^{-1}. Solid-state nuclear magnetic resonance (NMR) spectra were recorded on Bruker AM-400 NMR spectrometer. Atomic force microscope (AFM) experiments were carried out on Bruker Dimension ICON. Information of molecular structures and chemical bonds were determined by Fourier transform infrared (FT-IR, Nicolet 6700) in the rages of 500-4000 cm^{-1} and the operando FT-IR measurements were acquired at 1.1-1.7 *vs*. RHE in 1.0 M KOH electrolyte by Nicolet iS50 FT-IR (Thermo Fisher scientific). The N_2 sorption isotherms at 77 K were measured on Micrometrics ASAP 2020 Plus HD 88, and before testing, the samples were degassed at 120°C for 12 h under high vacuum to remove the guest molecules. The specific surface areas and pore size distributions of samples were calculated using the Brunauer-Emmet-Teller (BET) and the Barrett-Joyner-Halenda method, respectively. The C *K*-edge, O *K*-edge, and N *K*-edge X-ray absorption near-edge structure (XANES) spectra were performed at the Beamlines MCD-B in National Synchrotron Radiation Laboratory (NSRL). Operando differential electrochemical

mass spectrometry (DEMS) and isotope labeling data were measured by using QAS 100 device (Shanghai LingLu Instruments Company). According to the method described in the literature, the catalyst was labeled with ^{18}O-labeled 1.0 M KOH electrolyte by cyclic voltammetry. After the labeling was completed, the adsorbed ^{18}O on the catalyst surface was washed with a large amount of $H_2{}^{16}O$, and then tested in the 1.0 M KOH electrolyte which was prepared with $H_2{}^{16}O$ solution. The signals of gas products $^{32}O_2$, $^{34}O_2$ and $^{36}O_2$ were detected during the labeling process and the test process, respectively[4].

Computational Methods

The CASTEP (Cambridge Sequential Total Energy Package) module in the Materials Studio software was used to perform density functional theory (DFT) calculations for geometric optimization and energy relaxations. The plane-wave pseudopotential DFT methods with spin-polarized were adopted for the calculations. The exchange-correlation functional was described by the Generalized Gradient Approximation (GGA) and Perdew-Burke-Ernzerhof (PBE). The pseudopotential was described by Generated On The Fly (OTFG) ultrasoft, and relativistic treatment was conducted by Koelling-Harmon method. The cutoff of plane wave energy was set at 500 eV, and the convergence tolerance were set at 5×10^{-2} GPa, 3×10^{-2} eV·Å^{-1}, 1×10^{-5} eV·atom^{-1} and 1×10^{-3} Å for stresses, forces, energy and displacements, respectively.

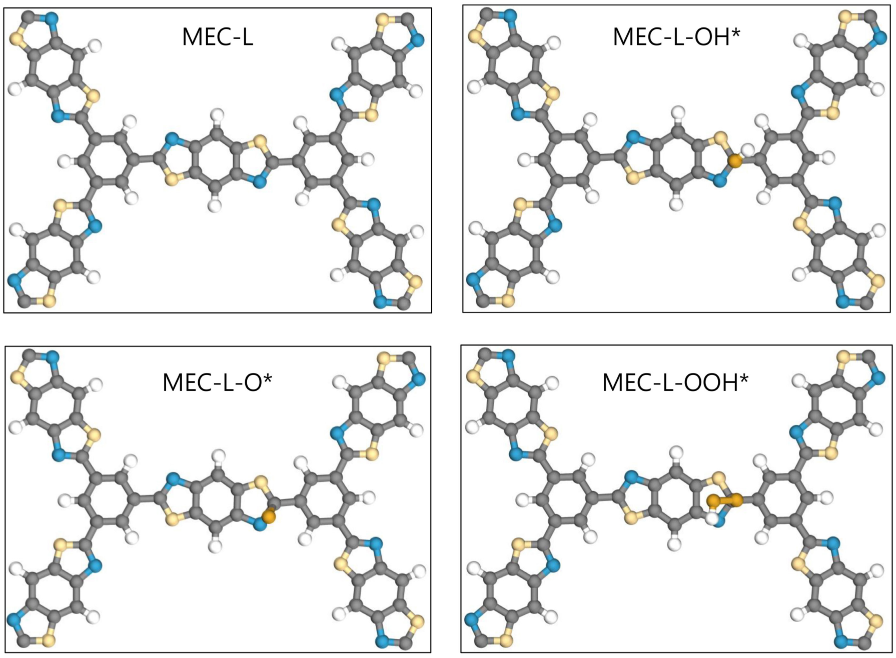

Figure S1. Structure models of MEC-L for key intermediates of AEM reaction pathway. The colors in the structure are represented: the gray ball represents carbon, the blue ball represents nitrogen, the yellow ball represents oxygen, and the white ball represents hydrogen.

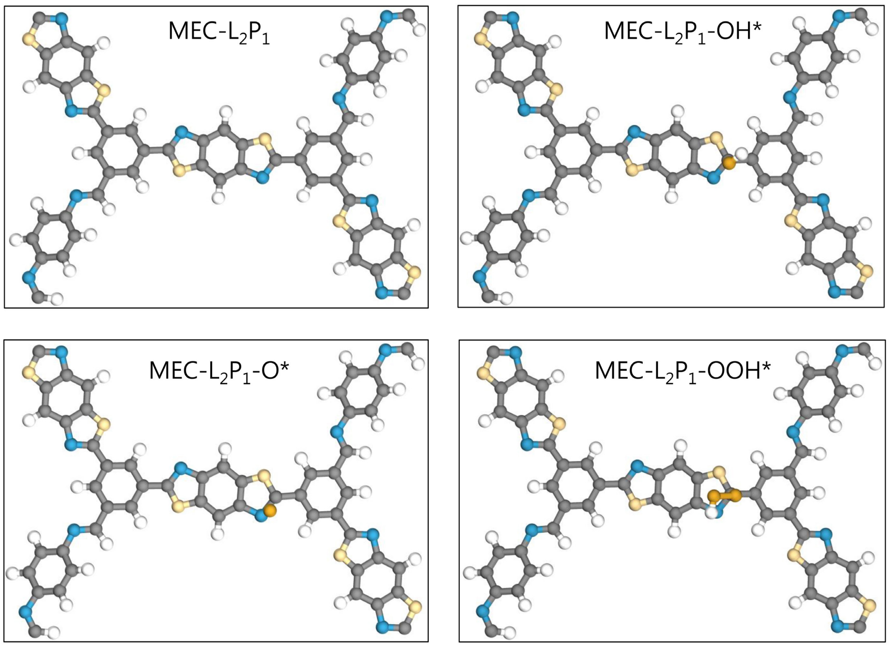

Figure S2. Structure models of MEC-L_2P_1 for key intermediates of AEM reaction pathway. The colors in the structure are represented: the gray ball represents carbon, the blue ball represents nitrogen, the yellow ball represents oxygen, and the white ball represents hydrogen.

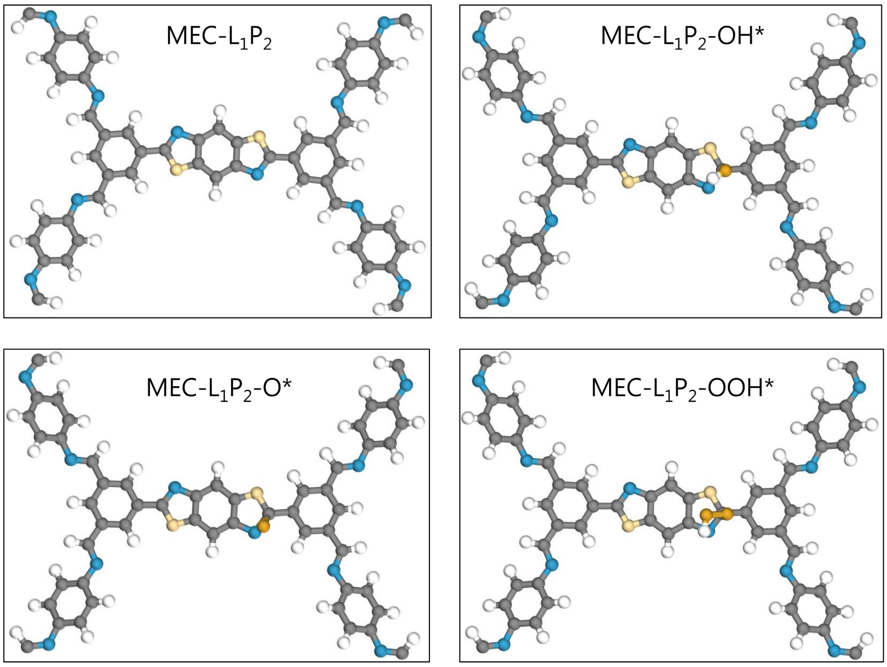

Figure S3. Structure models of MEC-L_1P_2 for key intermediates of AEM reaction pathway. The colors in the structure are represented: the gray ball represents carbon, the blue ball represents nitrogen, the yellow ball represents oxygen, and the white ball represents hydrogen.

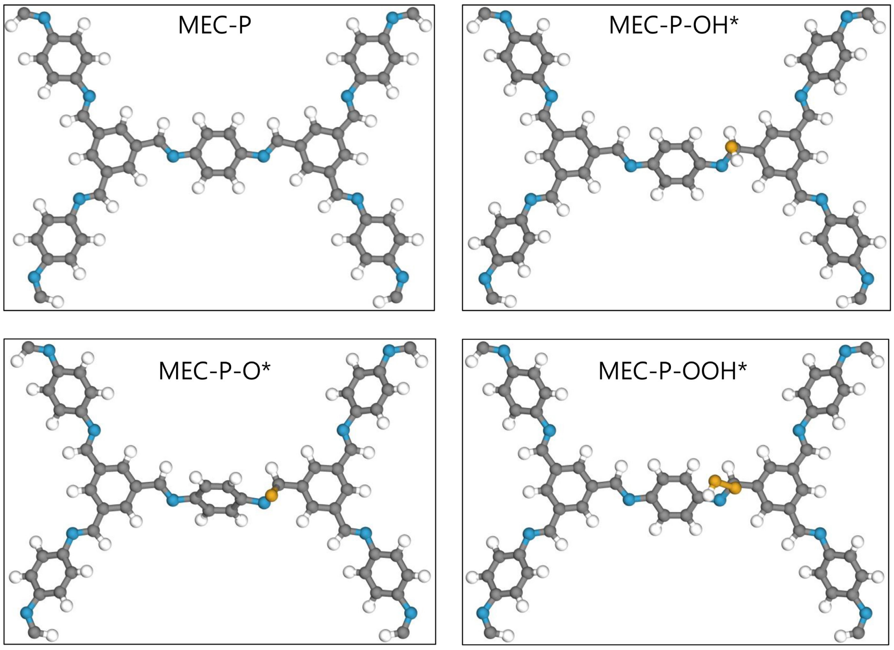

Figure S4. Structure models of MEC-P for key intermediates of AEM reaction pathway. The colors in the structure are represented: the gray ball represents carbon, the blue ball represents nitrogen, the yellow ball represents oxygen, and the white ball represents hydrogen.

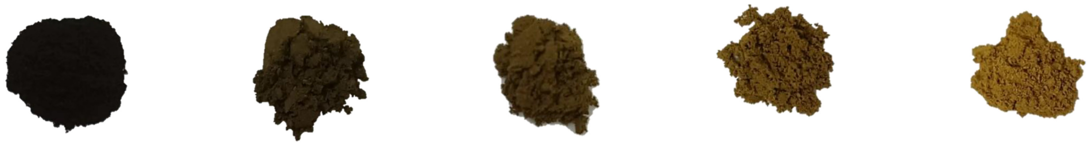

MEC-L MEC-L_2P_1 MEC-L_1P_1 MEC-L_1P_2 MEC-P

Figure S5. Optical photographs of catalysts. The color of the catalyst gradually transitions from black brown to earthy yellow, for example, the color of MEC-L_2P_1 is dark green, the color of MEC-L_1P_1 is brown-green, and the color of MEC-L_1P_2 is yellow-green.

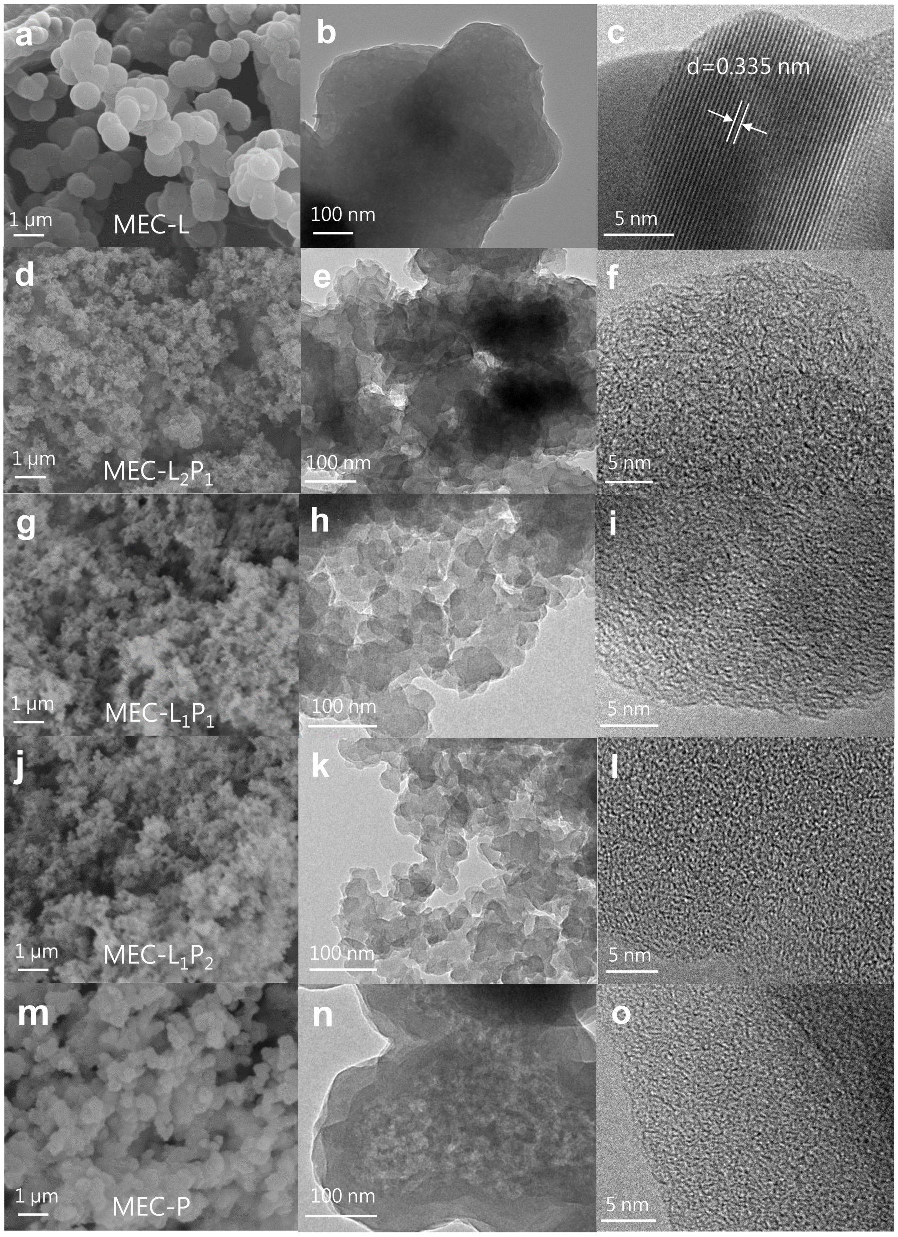

Figure S6. Morphological characterization of catalysts. SEM, TEM and HRTEM images of (a-c) MEC-L, (d-f) MEC-L_2P_1, (g-i) MEC-L_1P_1, (j-l) MEC-L_1P_2, (m-o) MEC-P.

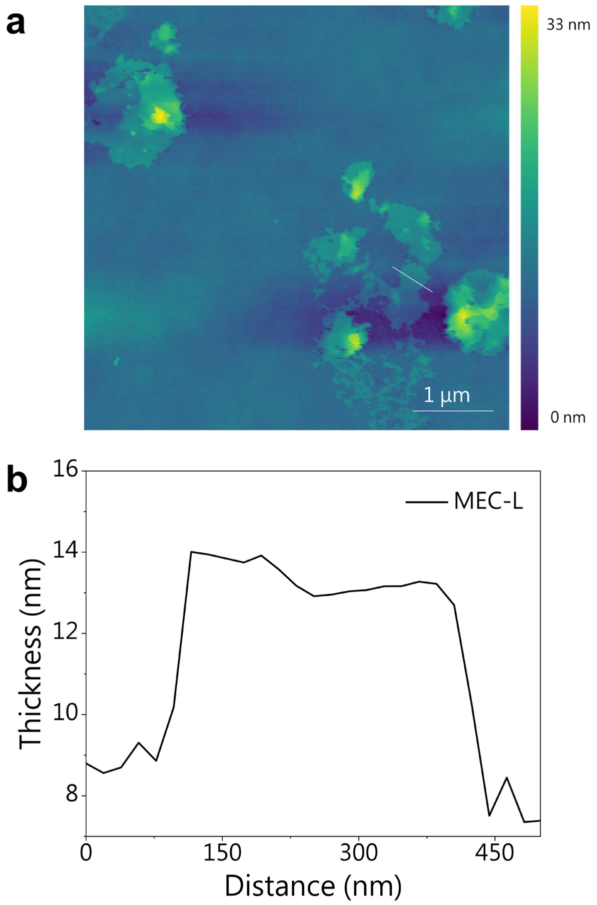

Figure S7. **AFM image and thickness of MEC-L.** (a) AFM image, (b) Thickness of MEC-L. The thickness of MEC-L is about 5.8±0.1 nm.

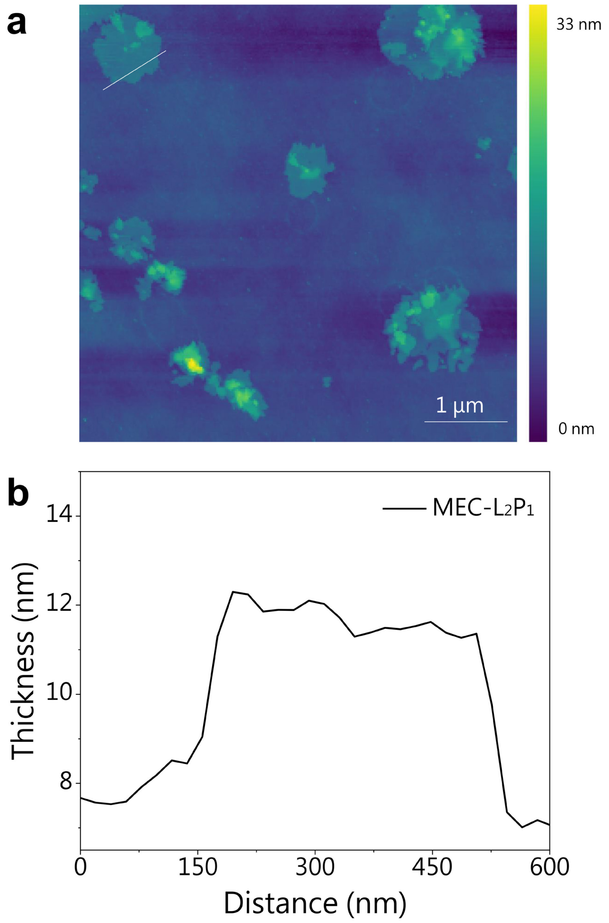

Figure S8. AFM image and thickness of MEC-L_2P_1. (a) AFM image, (b) Thickness of MEC-L_2P_1. The thickness of MEC- L_2P_1 is about 5.4±0.2 nm.

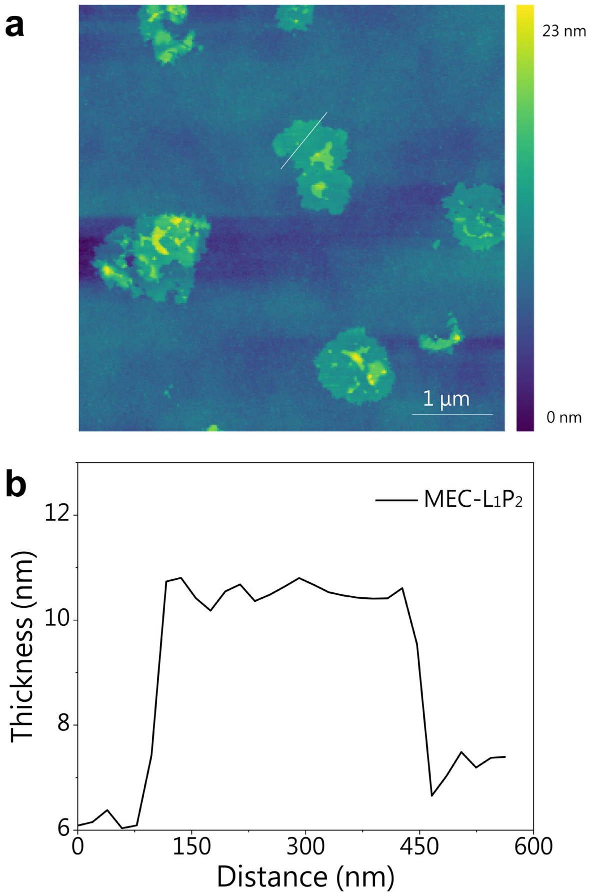

Figure S9. AFM image and thickness of MEC-L_1P_1. (a) AFM image, (b) Thickness of MEC-L_1P_2. The thickness of MEC- L_1P_2 is about 4.7 ± 0.1 nm.

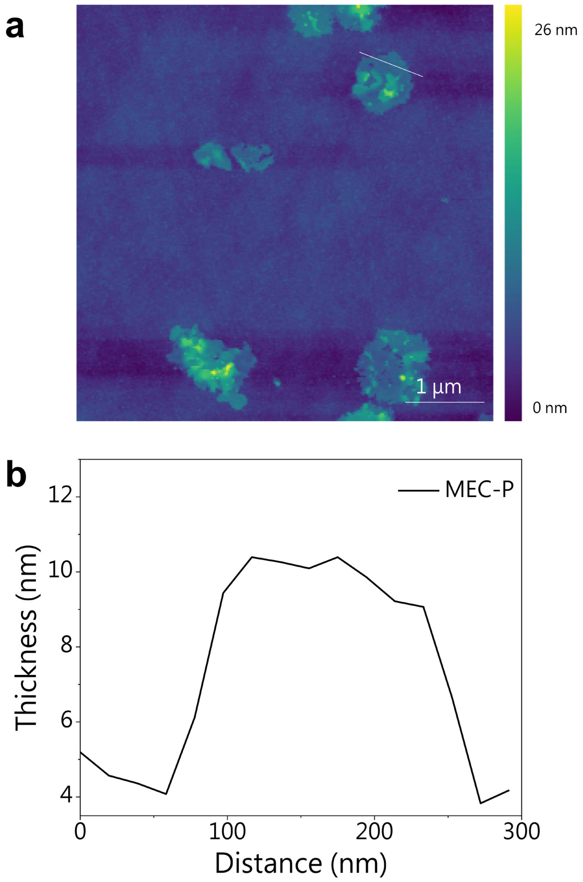

Figure S10. AFM image and thickness of MEC-P. (a) AFM image, (b) Thickness of MEC-P. The thickness of MEC- L_2P_1 is about 5.7 ± 0.1 nm.

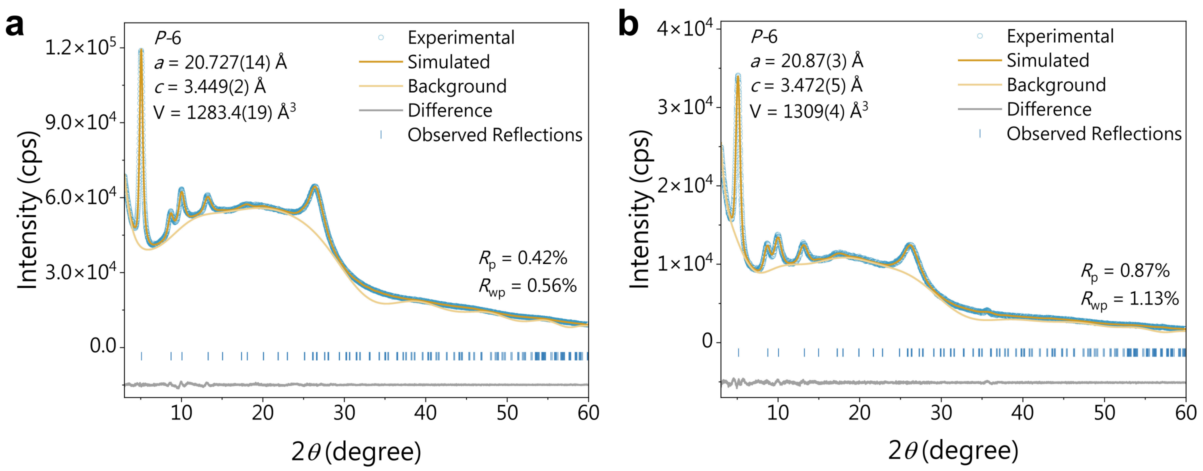

Figure S11. Observed PXRD pattern and refined modeling profile of catalysts. (a) Indexed experimental (blue), Pawley-refined (yellow) PXRD patterns of MEC-L, (b) Indexed experimental (blue), Pawley-refined (yellow) PXRD patterns of MEC-L_1P_1.

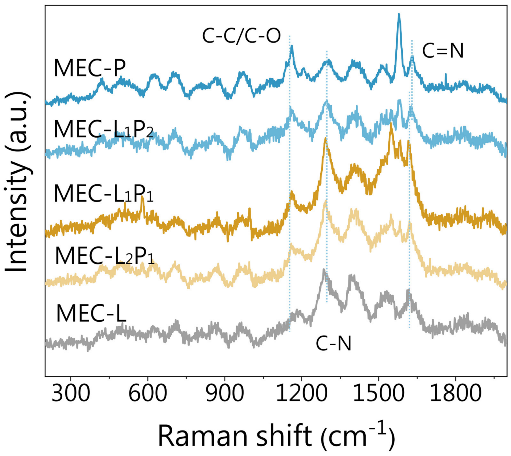

Figure S12. Raman spectra of catalysts. The presence of characteristic peaks D and G at about 1300 cm^{-1} and 1580 cm^{-1} in the MEC-L_xP_y sample indicates the formation of honeycomb crystal structure. In addition, the Raman peaks at 1162 cm^{-1}, 1341 cm^{-1} and 1635 cm^{-1} can be classified as the characteristic peaks of C−O/C−C, C−N and O−C=N, which confirms the synthesis of benzoxazole and imine structures.

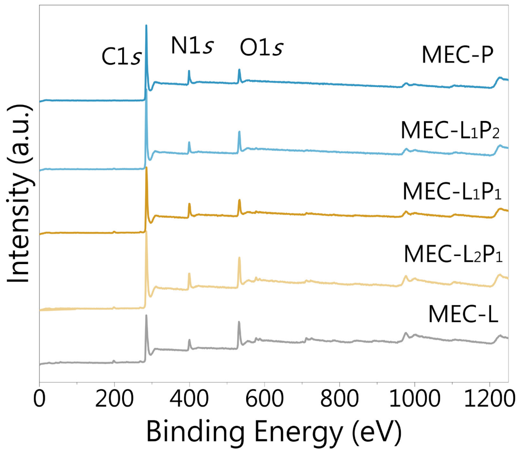

Figure S13. XPS survey scans of catalysts. With the increase of PPD feeding ratio, the peak intensity of O 1*s* decreased, and the oxygen content decreased from 34.02% (MEC-L) to 14.41% (MEC-P).

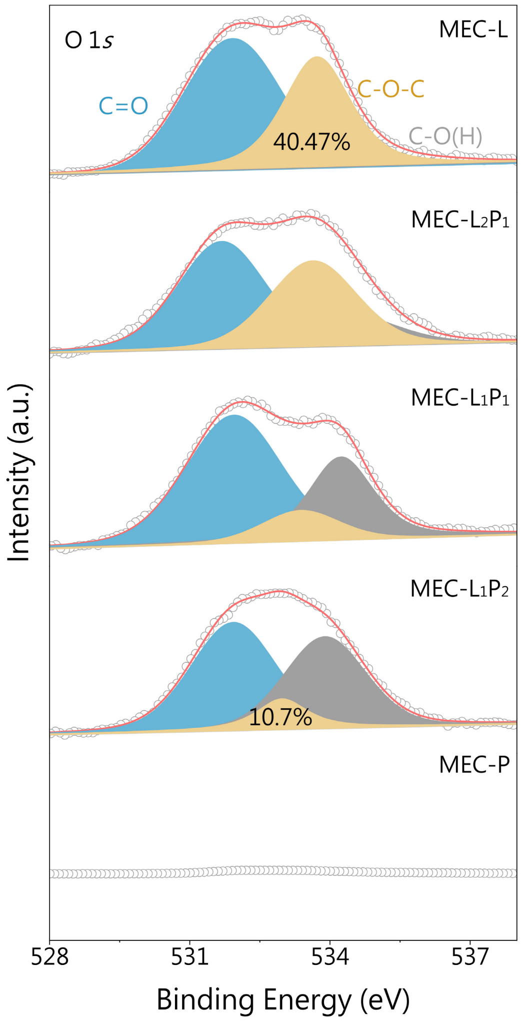

Figure S14. High-resolution O 1*s* XPS spectra of catalysts. With the increase of the proportion of PPD, the C−O−C peak shifted to the direction of low binding energy and the percentage content gradually decreased (from 40.47% to 10.70%), which was speculated to be due to the decrease of the content of benzoxazole rings in the structure.

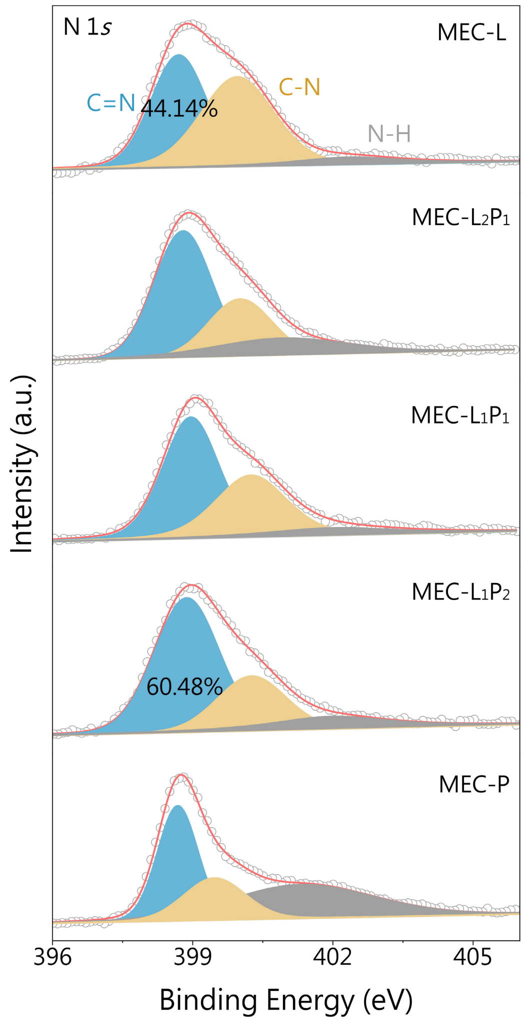

Figure S15. High-resolution N 1*s* XPS spectra of catalysts. The C=N peak shifted towards the direction of high binding energy, and because the increase of imine bonds, the percentage increased gradually (from 44.14% to 60.48%).

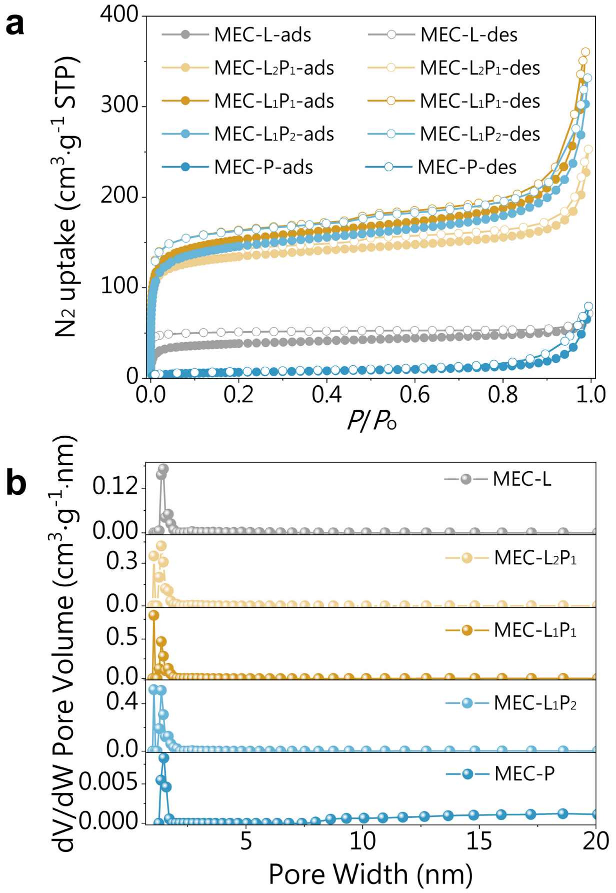

Figure S16. N_2 sorption isotherms and pore size distribution of catalysts. (a) 77 K nitrogen adsorption isotherms of MEC-L_xP_y. (b) Pore size distribution of MEC-L_xP_y. MEC-L_xP_y have micropores with pore sizes between 0.5 and 1.5 nm.

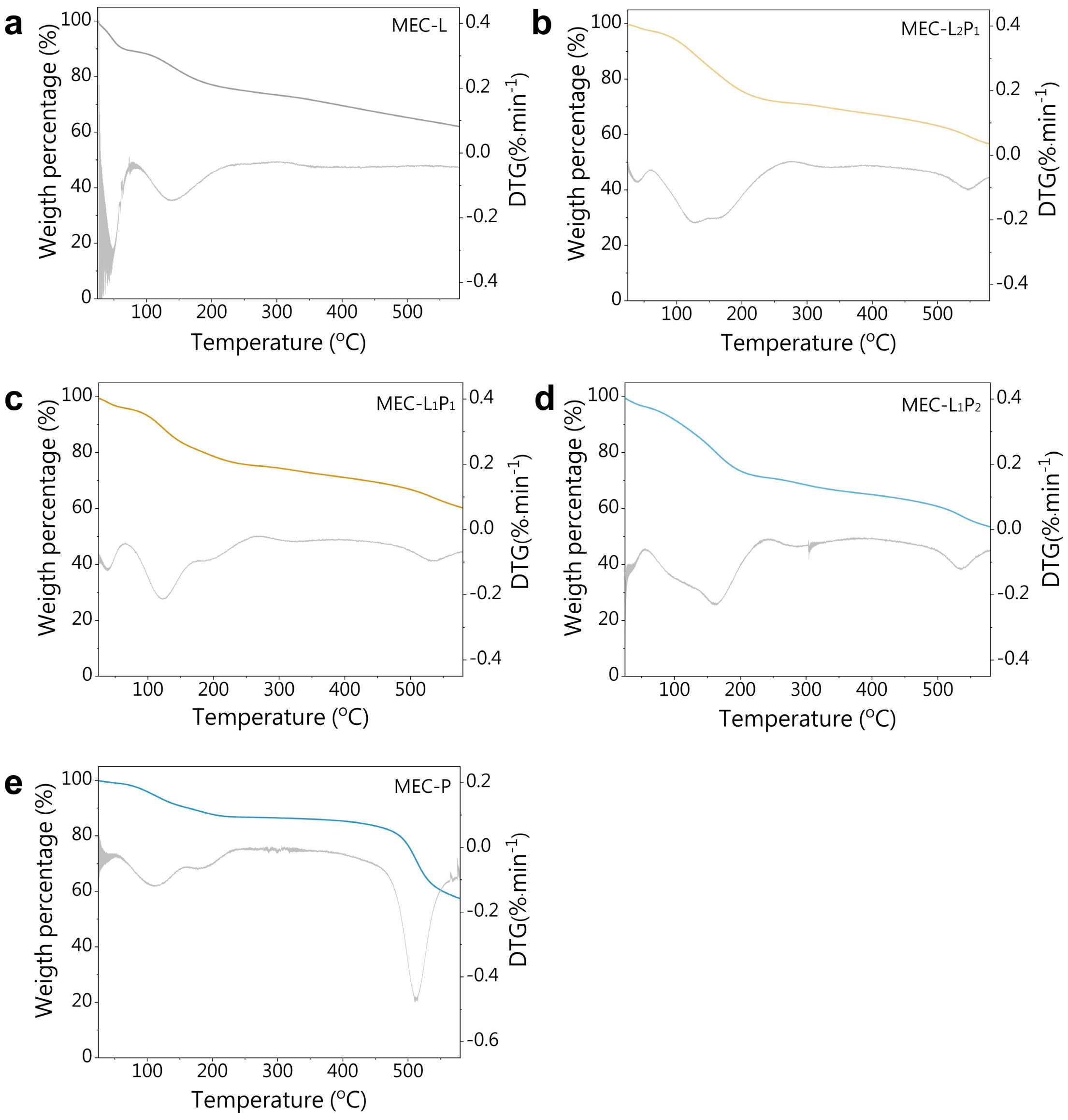

Figure S17. TG curves and DTG curves of catalysts. TG curves and DTG curves of (a) MEC-L, (b) MEC-L_2P_1, (c) MEC-L_1P_1, (d) MEC-L_1P_2, and (e) MEC-P in N_2 atmosphere, respectively. MEC-L_xP_y have excellent thermal stability and can withstand temperatures above 400°C.

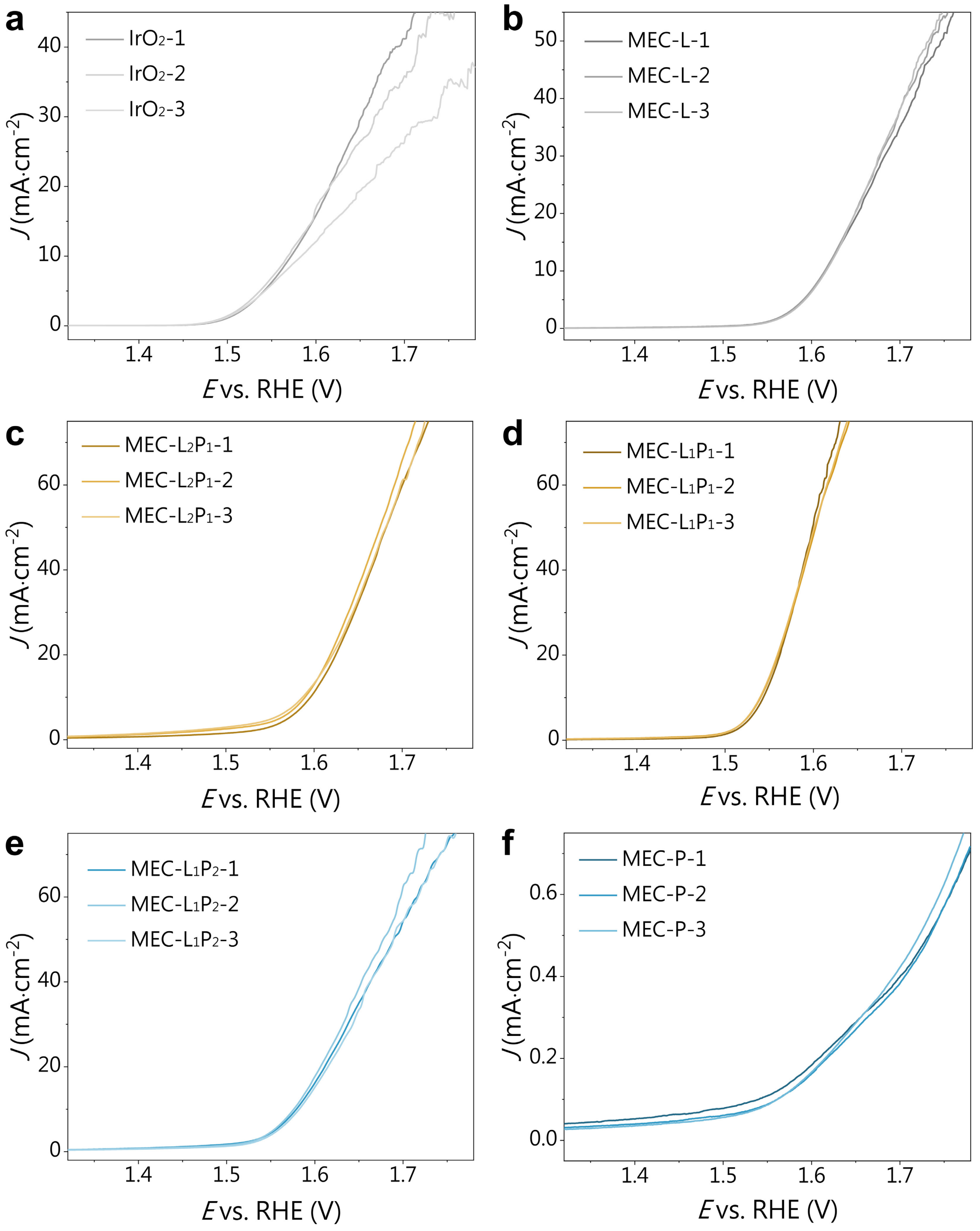

Figure S18. LSV curves of catalysts. Each sample tested 3 times on different GCE. When the current density is 10 mA·cm^{-2}, the overpotentials of IrO_2, MEC-L, MEC-L_2P_1, MEC-L_1P_1, MEC-L_1P_2 are 344.3±7.8 mV, 386.0±1.0 mV, 361.0±4.6 mV, 311.0±1.7 mV, and 349.0±3.0 mV, respectively.

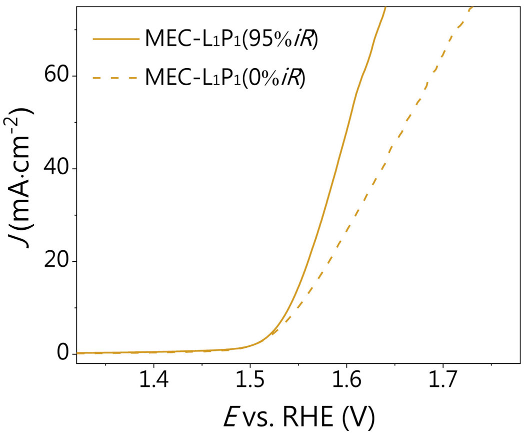

Figure S19. LSV curves of MEC-L_1P_1 with 95% and 0% *iR* compensation. LSV curves were obtained with 95% and 0% *iR* compensation in O_2-saturated 1.0 M KOH at room temperature.

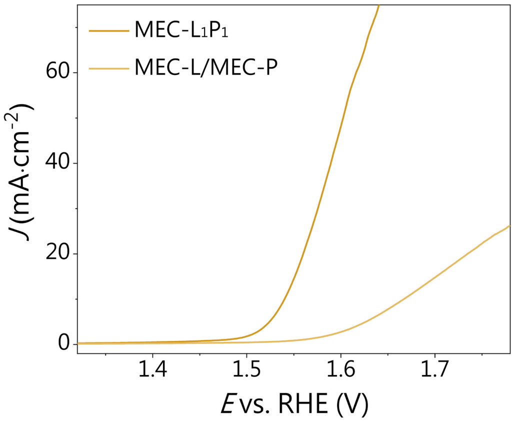

Figure S20. LSV curves of MEC-L_1P_1 and MEC-L/MEC-P. The OER performance of MEC-L_1P_1 is much better than that of the mechanically mixed MEC-L/MEC-P, suggesting that the coexistence of benzodioxazole and benzodiimide in the identical molecular framework can enhance intrinsic activity.

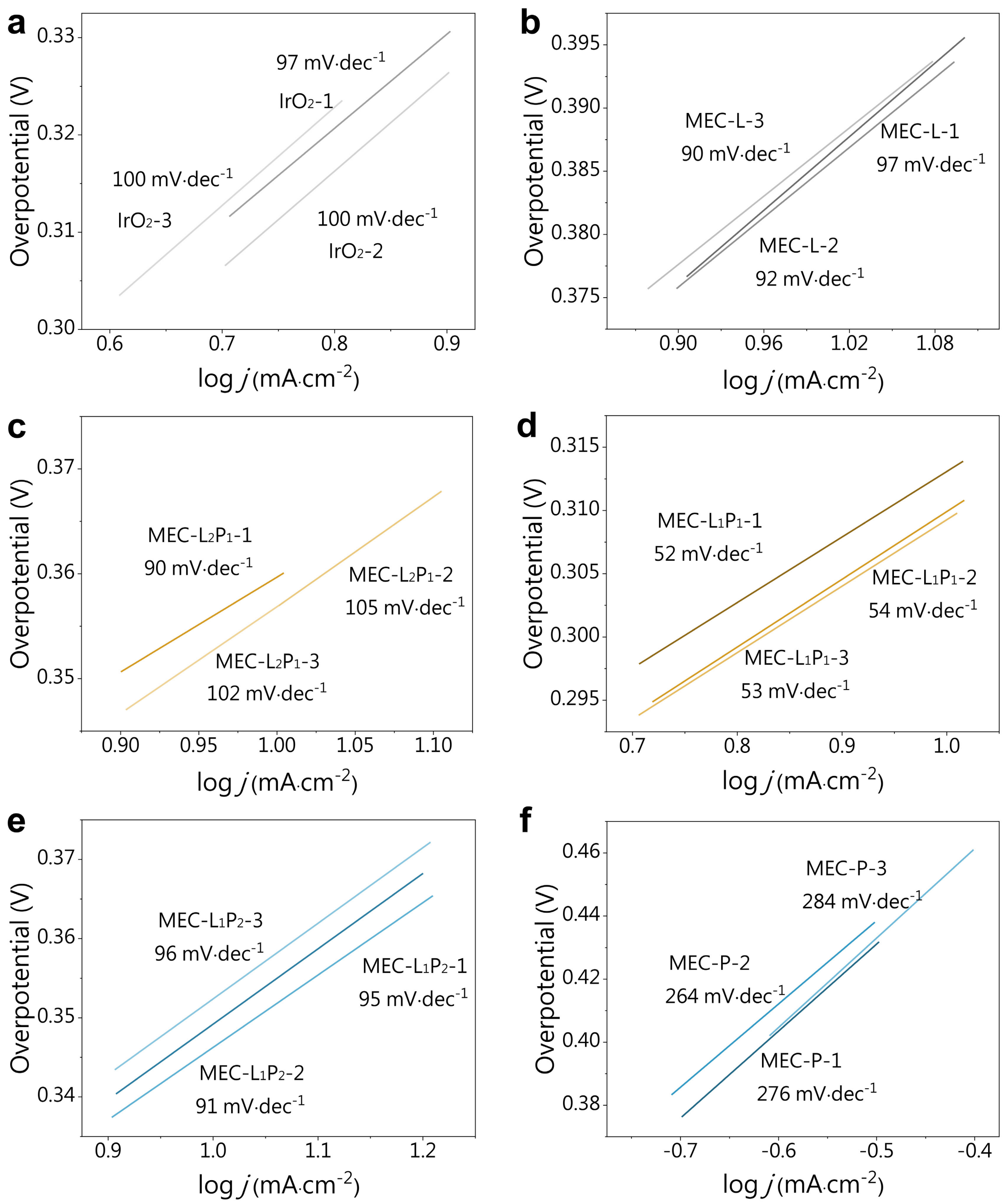

Figure S21. Tafel slopes of catalysts. The values of Tafel slope were obtained by fitting the gentle linear part of the Tafel plot. According to the data of the three tests in Figure S17, the Tafel slopes of MEC-L, MEC-L_2P_1, MEC-L_1P_1, MEC-L_1P_2 can be estimated to be 99.0±1.7 $mV\cdot dec^{-1}$, 93.0±3.6 $mV\cdot dec^{-1}$, 91.7±8.6 $mV\cdot dec^{-1}$, 53.0±1.0 $mV\cdot dec^{-1}$, and 94.0±2.6 $mV\cdot dec^{-1}$, respectively.

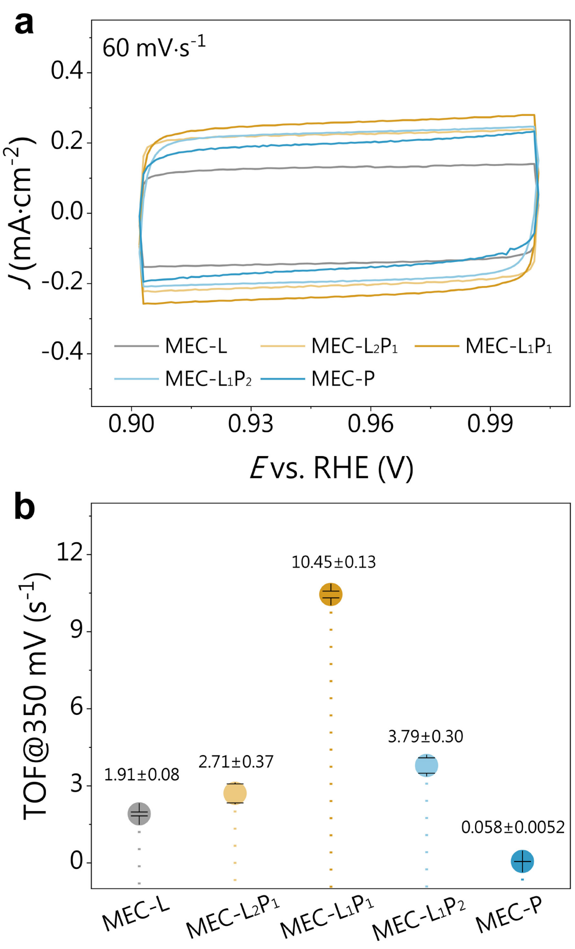

Figure S22. Redox area curve and TOF values of catalysts. (a) CV scan (scan rate is 60 mV $\cdot s^{-1}$) of catalysts. (b) TOF values of catalysts. When the CV scanning rate is 60 mV$\cdot s^{-1}$, the area of CV curves tested in the non-Faraday interval test are used to estimate the TOF values of various catalysts. When the overpotential of the catalyst is 350 mV, the maximum TOF value of MEC-L_1P_1 is 10.45±0.13 s^{-1}.

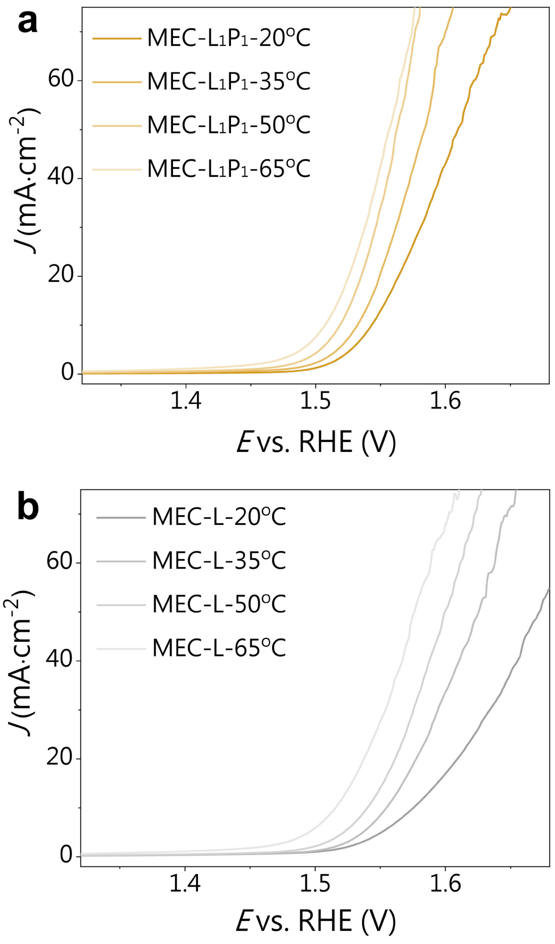

Figure S23. LSV curves of catalysts tested at different temperatures. OER polarization curves of (a) MEC-L_1P_1 and (b) MEC-L acquired with 95% *iR* compensation in O_2-saturated 1.0 M KOH aqueous electrolyte with a scan rate of 5 $mV \cdot s^{-1}$ at different temperatures.

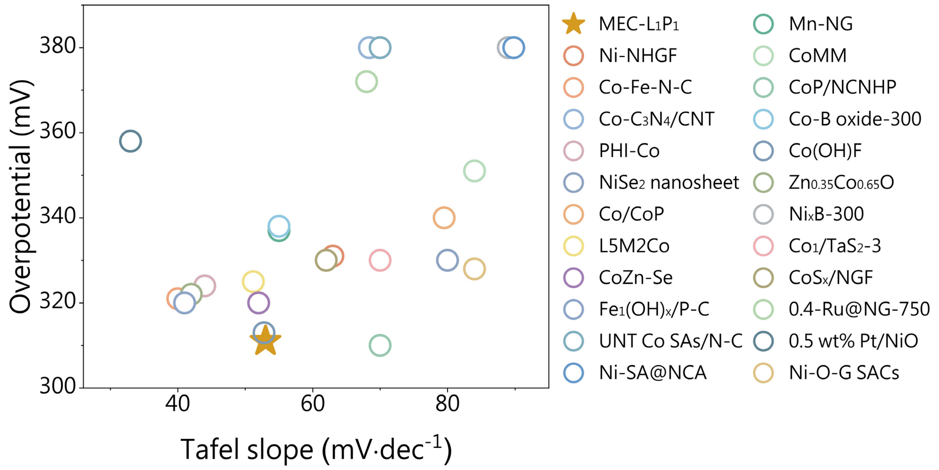

Figure S24. Comparison of electrocatalytic performance of catalysts (metal-based electrocatalysts). Comparison of OER performances of MEC-L_1P_1 and the recently reported metal-based electrocatalysts on GCE in 1.0 M KOH.

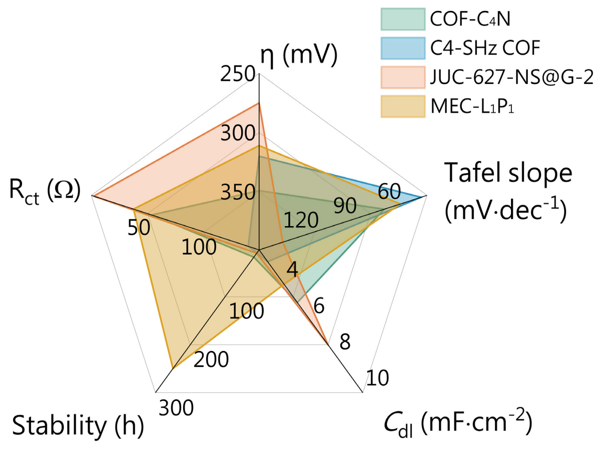

Figure S25. Comparison of electrocatalytic performance of catalysts (metal-free COF). Comparison of OER performances of MEC-L_1P_1 and the reported metal-free covalent organic framework electrocatalysts.

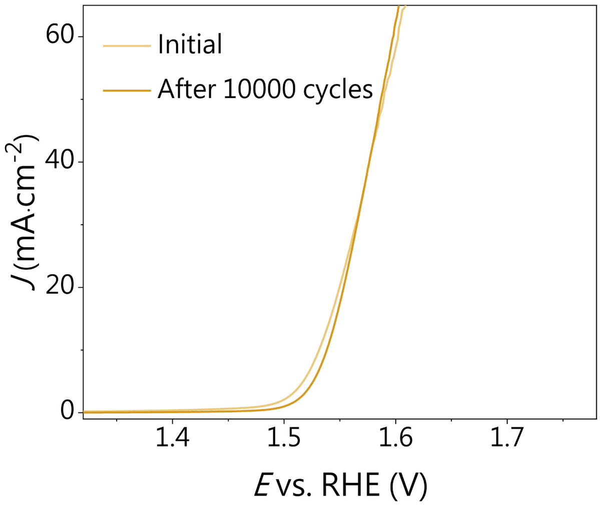

Figure S26. LSV curves of MEC-L_1P_1 before and after 10000 cycles of CV scan on GCE. After 10000 cycles CV scanning, there was no significant difference in the electrocatalytic performance of the catalyst, indicating that MEC-L_1P_1 had good electrochemical stability.

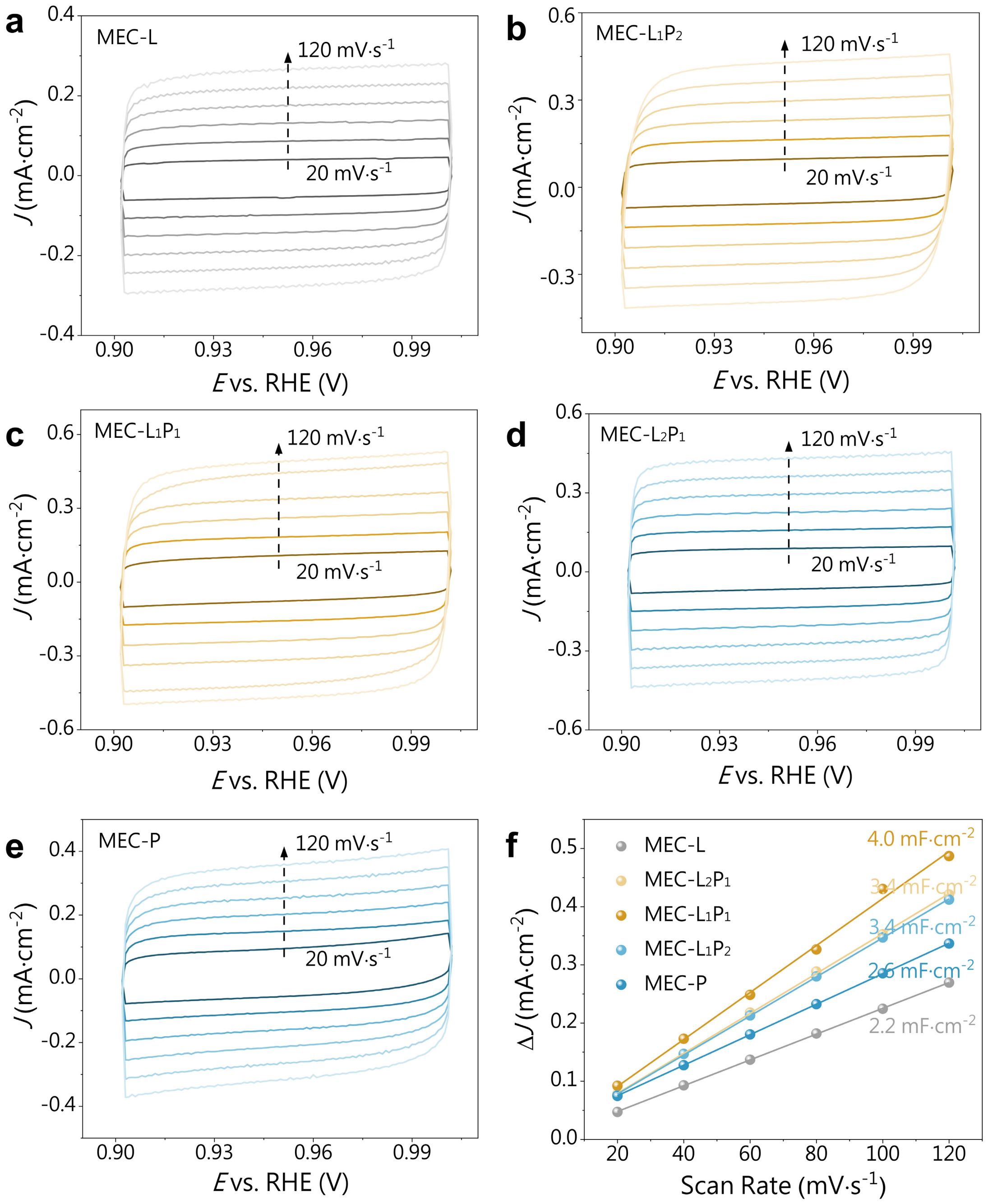

Figure S27. CV curves of catalysts on GCE and C_{dl} comparison of catalysts. CVs of (a) MEC-L, (b) MEC-L_2P_1, (c) MEC-L_1P_1, (d) MEC-L_1P_2, and (e) MEC-P in 1.0 M KOH solution at different scan rates (20, 40, 60, 80, 100, 120 mV·s^{-1}) on GCE at room temperature. (f) C_{dl} comparison of MEC-L, MEC-L_2P_1, MEC-L_1P_1, MEC-L_1P_2, and MEC-P with different CV scan rates.

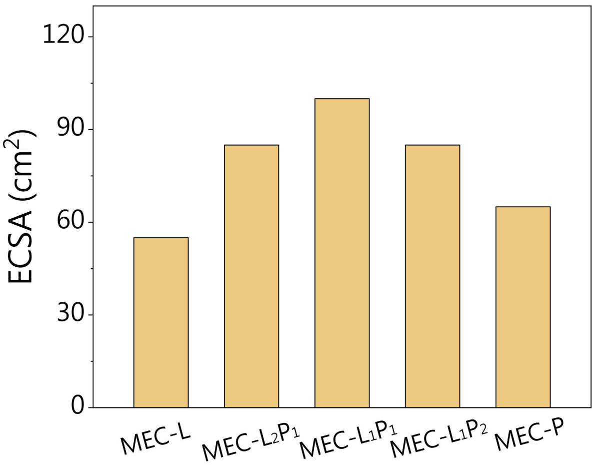

Figure S28. ECSA values of catalysts. The value of C_s is 0.04 mF·cm^{-2}, then the ECSA value increases with the increase of *p*-phenylenediamine doping amount. When the doping amount of *p*-phenylenediamine is 50%, the ECSA value is the largest, and then the doping amount of *p*-phenylenediamine continues to increase, and the ECSA value decreases.

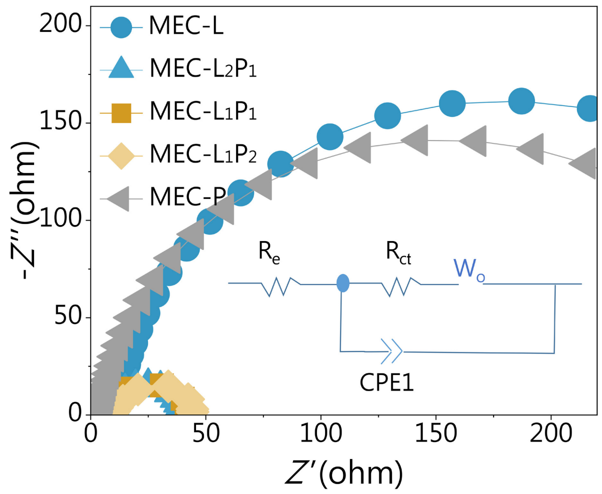

Figure S29. Electrochemical impedance profiles of MEC-L$_x$P$_y$. The EIS of catalysts was measured at a voltage of 1.5 V vs. RHE.

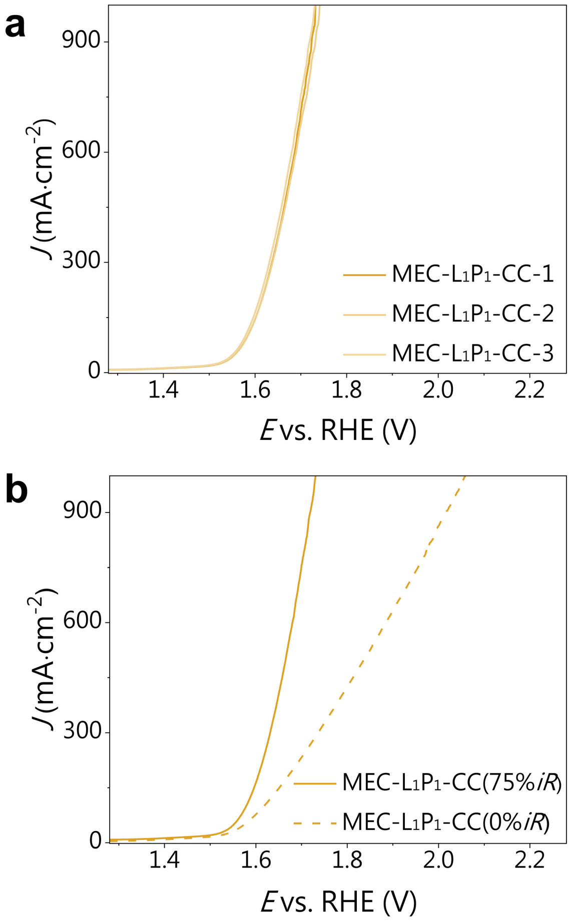

Figure S30. LSV curves of catalysts supported on carbon cloth. (a) LSV curves of MEC-L_1P_1 supported on carbon cloth. Each sample tested 3 times on different carbon cloth. (b) LSV curves of MEC-L_1P_1 supported on carbon cloth with 75% and 0% *iR* compensation.

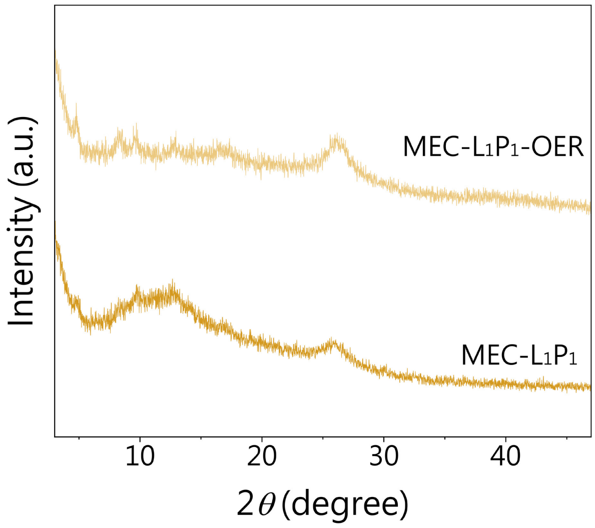

Figure S31. PXRD patterns of catalysts before and after electrocatalytic test. After electrochemical reaction, the characteristic peaks of MEC-L_1P_1 did not significantly changes, indicating that the structural integrity of MEC-L_1P_1 could be maintained.

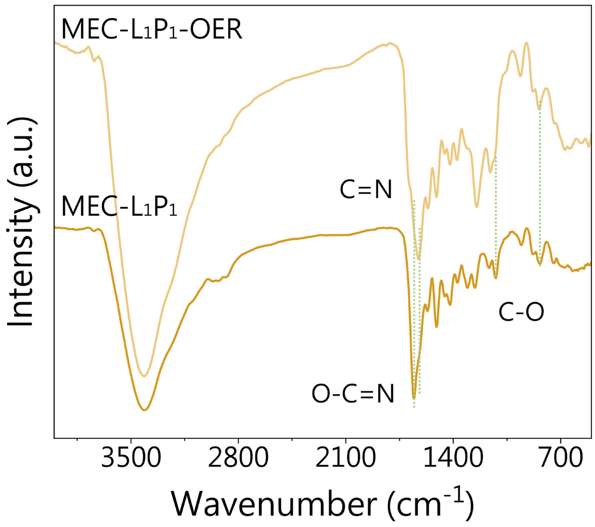

Figure S32. FTIR spectra of catalysts before and after electrocatalytic test. After electrochemical reaction, the infrared characteristic peak of MEC-L_1P_1 did not change significantly, indicating that the structural integrity of MEC-L_1P_1 could be maintained.

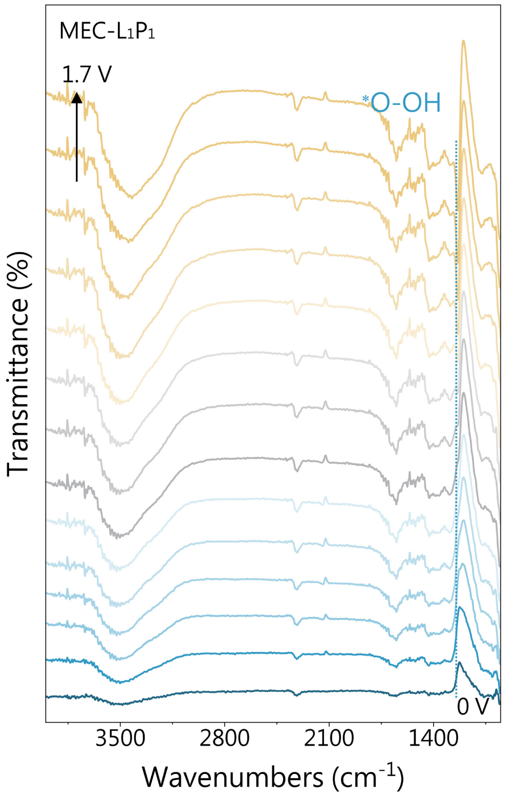

Figure S33. Potential-dependent *operando* ATR-SEIRAS of MEC-L$_1$P$_1$. Potential-dependent *operando* ATR-SEIRAS was measured in the potential range of 1.1-1.7 V vs. RHE. The appearance of an infrared signal peak attributed to *O–OH at 1224 cm^{-1} indicates that the OER process of MEC-L$_1$P$_1$ follows the AEM pathway.

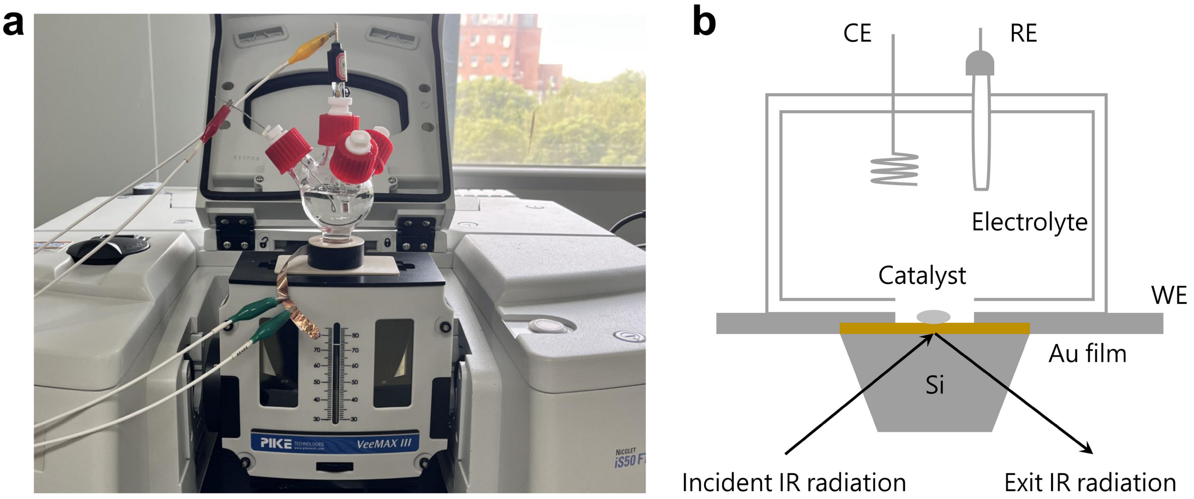

Figure S34. *Operando* ATR-SEIRAS system configuration. (a) A photograph of the *operando* ATR-SEIRAS system configuration, (b) The schematic diagram of *Operando* ATR-SEIRAS system configuration.

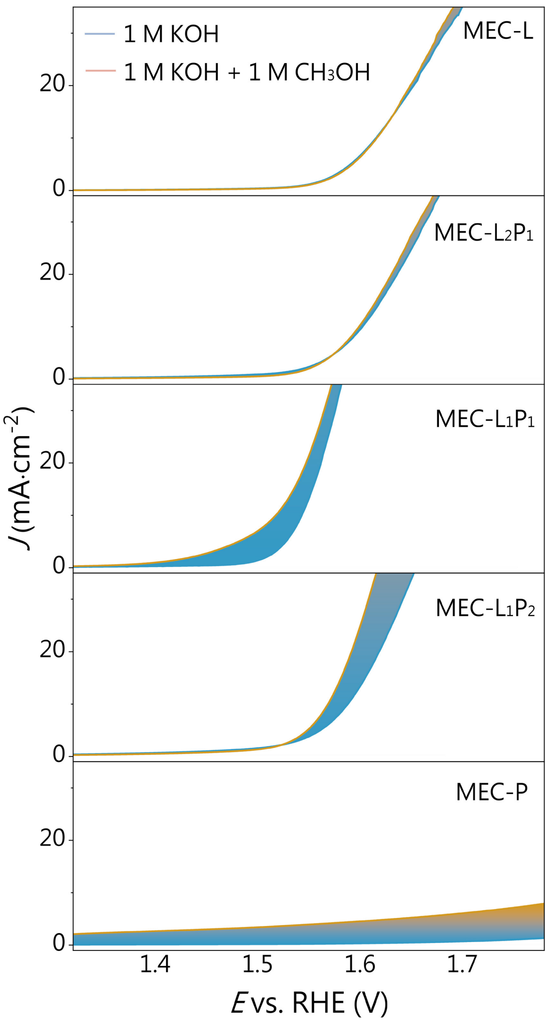

Figure S35. The LSV curve of catalysts. The LSV curve of MEC-L_xP_y were recorded in 1.0 M KOH and 1.0 M KOH + CH_3OH, and all of the LSV curves were obtained with 95% *iR* compensation. Methanol oxidation reaction (MOR) experiments revealed that MEC-L_1P_1 exhibits moderate surface coverage of OH.

Table S1. Comparison of OER performance of MEC-L_1P_1 with reported metal-free electrocatalysts in 1.0 M KOH.

**Catalysts**	**η_{10} (mV)**	**Tafel (mV·dec^{-1})**	**Support**	**Q (C·cm^{-2})**	**Reference**
			**COF**		
**MEC-L_1P_1**	**311±2**	**53±1**	**GCE**	**/**	**This work**
**MEC-L_1P_1**	**341±14 (η_{200})**	/	CC	**90000**	**This work**
COF-C_4N	349	64	GCE	720	ACS Energy Lett. 2019[5]
C4-SHz COF	320	39	GCE	396	ACS Catal. 2020[6]
TAPTt-COF (CC-3)^a	389	101	GCE	/	ACS Nano. 2021[7]
JUC-627-NS@G-2	275	137	GCE	540	Chem. Eng. J. 2023[8]
F-NPC^a	420	181	GCE	/	J. Colloid Interface Sci. 2023[9]
JUC-630	400	104	GCE	97.2	Acta Phys. Chim. Sin. 2023[10]
PPPI^a	470	75	CC	/	Angew. Chem. Int. Ed. 2018[11]
			**POP**		
THT-PyDAN	283	81	CC	/	Adv. Mater. 2024[12]
TAT-TFBE	314	42	CC	1080	Adv. Mater. 2024[13]
Porphvlar	700	131	CP	2160	Chem. Sci. 2022[14]
EPOP	440	169	CP	216	ACS Omega. 2018[15]
MCAC^a	330	60	GCE	/	J. Colloid Interface Sci. 2021[4]
P-TzTz	583	110	GCE	216	Mater. Today Chem. 2023[16]
CPF-2	328	89	GCE	1800	ACS Appl. Mater. Interfaces. 2023[17]
CPF-1	488	56	GCE	1800	ACS Appl. Mater. Interfaces. 2023[17]
			**Other metal-free electrocatalysts**		
PDDA@CNTs93^a	370	76	GCE	/	Energy Environ. Sci. 2018[18]
g-C_3N_4 NS–CNT	370	83	GCE	/	Angew. Chem. Int. Ed. 2014[19]
g-C_3N_4 –GO^a	539	69	GCE	/	Chemsuschem. 2014[20]
N-graphene/CNT^a	420	/	GCE	/	Angew. Chem. Int. Ed. 2014[21]
Defective graphene^a	440	/	GCE	/	Adv. Mater. 2016[22]
NG-CNT^a	360	141	GCE	/	Adv. Mater. 2014[23]

N-SMCTs@N-rGO	351	/	GCE	3600	Chem. Eng. J. 2022[24]
5%-BCNONF	403	73	GCE	360	Nano Energy. 2021[25]
N-GP	330	55	GCE	10800	J. Am. Chem. Soc. 2022[26]
S-C_3N_4/CNT[a]	440	81	GCE	/	ACS Appl. Mater. Interfaces. 2022[27]
D/G-HASP[a]	310	65	GCE	/	J. Mater. Chem. A. 2021[28]
CNTs-NC-CCC[a]	380	131	GCE	/	Appl Catal B: Environ. 2022[29]
N-doped mesoporous graphene framework[a]	430	/	GCE	/	Catal. Today 2018[30]
GH-$BGQD_2$[a]	370	70	GCE	/	Adv. Energy Mater. 2019[31]
PNGF(op)[a]	320	/	GCE	/	Energy Environ. Sci. 2017[32]
DAP_1-$NDA_{0.5}$-$DBU_{0.5}$[a]	350	108.49	GCE	/	Appl. Mater. Today 2020[33]
NL-CNT-3[a]	360	59	GCE	/	ChemCatChem 2019[34]
VGNO[a]	490	323.4	GCE	/	Adv. Sci. 2022[35]
N-OMC_2[a]	519	/	GCE	/	J. Energy Chem. 2017[36]
NPCGF-6:4[a]	461	/	GCE	/	ChemCatChem 2017[37]
EBP@NG(1:8)	310	89	GCE	432	J. Am. Chem. Soc. 2019[38]
g-CN	316	125	GCE	/	ChemCatChem 2019[39]
G–P	330	62	GCE	/	Chem. Commun. 2016[40]
G-NC(1.5)	330	52.6	GCE	/	ChemSusChem 2019[41]
PANI@CNTs75	331	85	GCE	198	Chem Asian J. 2020[42]
G-EX-ST	349	44	GCE	/	Chem. Commun. 2019[43]
LIG-O	360	49	GCE	/	Adv. Mater. 2018[44]
S-doped BP	410	75	GCE	/	Nanotechnology 2019[45]
FPQDs[b]	430	48	GCE	/	Acs Nano. 2018[46]
N-HC@G-900	350	88	GCE	270	Angew. Chem. Int. Ed. 2018[47]
PAA@CNTs90	344	99	GCE	/	Energy Environ. Sci. 2017[48]
6,13-PQ-modified OLC	350	62	GCE	360	J. Am. Chem. Soc. 2018[49]
echo-MWCNTs	350	41	GCE	48.6	J. Am. Chem. Soc. 2015[50]
GO-PANi-FP	390	136	GCE	/	Angew. Chem. Int. Ed. 2016[51]
N-GRW	360	47	GCE	864	Sci. Adv.2016[52]

SHG	330	71	GCE	720	Adv. Mater. 2017[53]
S, S'-CNT1000°C	350	95	GCE	/	Adv. Energy Mater. 2016[54]
PEMAc@CNTs90	298	52	GCE	135	Energy Environ. Sci. 2017[48]
O-CNT	360	48	GCE	432	ACS Energy Lett. 2017[2a]
B, N-Carbon	380	84	GCE	/	Adv. Sci. 2018[55]
NDGs-800	450	132	GCE	/	ACS Energy Lett. 2018[56]
P-TzTz-CNS800	347	129	GCE	1620	Mater. Today Chem. 2023[16]
N, B, F doped PCNFs	280	83	GCE	1800	J. Mater. Chem. A. 2024[57]
NPCNF-O	326	178	GCE	/	ACS Catal. 2022[58]
PANa	316	42	CC	/	Nat. Commun. 2023[59]
p-FGDY/CC	460	128	CC	324	Angew. Chem. Int. Ed. 2019[60]
PA-PPy/CC	340	55	CC	/	Angew. Chem. Int.Ed. 2019[61]
PS-CNF[a]	320	89	CC	/	Nanoscale Horiz. 2017[62]
Activated CC	360	52	CC	/	Adv. Energy Mater. 2019[63]
NiD-PCC	360	98	CC	1008	Energy Environ. Sci. 2016[64]
P-CC	450	/	CC	198	Adv. Mater. 2017[65]
N, OVAGNs/CC	351	38	CC	1440	J. Mater. Chem. A 2018[66]
CC-9NH_3-3h	298	62	CC	3600	Appl. Catal. B: Environ. 2021[67]
P-C_3N_4/CC	400 (η_{100})	62		/	Angew. Chem. Int.Ed. 2015[68]
Oxidized carbon cloth	477 (η_{100})	82	CC	/	Chem. Commun. 2015[69]
G-C_3N_4 film	414 (η_{100})	128		/	Adv. Sci. 2015[70]
N, S-doped graphite foil (NSGF)	346	78	GFLs	/	Adv. Energy Mater. 2016[71]

[a]The electrolyte is 0.1 M KOH electrolyte. [b] The electrolyte is 1.0 M NaOH. GCE = glassy carbon electrode; CC = carbon cloth; CP = carbon paper; POP = porous organic polymer.

Table S2. Comparison of OER performance of MEC-L_1P_1 with reported metal-based electrocatalysts on GCE in 1.0 M KOH.

**Catalysts**	**η_{10} (mV)**	**Tafel (mV·dec^{-1})**	**Reference**
**MEC-L_1P_1**	**311±2**	**53±1**	**This work**
Mn-NG	337	55	Nat. Catal. 2018[72]
Ni–NHGF	331	63	Nat. Catal. 2018[73]
CoMM	351	84	J. Am. Chem. Soc. 2023[74]
Co-Fe-N-C	321	40	J. Am. Chem. Soc. 2019[75]
CoP/NCNHP	310	70	J. Am. Chem. Soc. 2018[76]
Co–C_3N_4/CNT	380	68.4	J. Am. Chem. Soc. 2017[77]
Co-B oxide-300	338	55	Angew. Chem. Int. Ed. 2022[78]
NiCoP/C	350	/	Angew. Chem. Int. Ed. 2017[79]
PHI-Co	324	44	Adv. Mater. 2020[80]
Co(OH)F	313	52.8	Adv. Mater. 2017[81]
$NiSe_2$ nanosheets	330	80	Adv. Mater. 2017[82]
$Zn_{0.35}Co_{0.65}O$	322	42	Adv. Energy Mater. 2019[83]
Co/CoP	340	79.5	Adv. Energy Mater. 2017[84]
Ni_xB-300	380	89	Adv. Energy Mater. 2017[85]
L5M2Co	325	51.2	Adv. Funct. Mater. 2021[86]
Co_1/TaS_2-3	330	70	ACS Nano 2021[87]
CoZn-Se	320	52	ACS Nano 2019[88]
CoS_x/NGF	330	62	ACS Nano 2018[89]
$Fe_1(OH)_x$/P-C	320	41	Nano Lett. 2021[90]
0.4-Ru@NG-750	372	68	ACS Catal. 2019[91]
UNT Co SAs/N-C	380	70	Nano Energy 2019[92]
0.5 wt% Pt/NiO	358	33	Chem. Sci., 2018[93]
Ni-SA@NCA	380	89.8	Chem. Eng. J. 2021[94]
Ni–O–G SACs	328	84	J. Energy Chem. 2019[95]

References

[1] P.-F. Wei, M.-Z. Qi, Z.-P. Wang, S.-Y. Ding, W. Yu, Q. Liu, L.-K. Wang, H.-Z. Wang, W.-K. An, W. Wang, *J. Am. Chem. Soc.* **2018**, 140, 4623.

[2] a)L. Li, H. Yang, J. Miao, L. Zhang, H.-Y. Wang, Z. Zeng, W. Huang, X. Dong, B. Liu, *ACS Energy Lett.* **2017**, 2, 294; b)A. Li, S. Kong, C. Guo, H. Ooka, K. Adachi, D. Hashizume, Q. Jiang, H. Han, J. Xiao, R. Nakamura, *Nat. Catal.* **2022**, 5, 109.

[3] A. Sadhukhan, A. Karmakar, K. Koner, S. Karak, R. K. Sharma, A. Roy, P. Sen, K. K. Dey, V. Mahalingam, B. Pathak, S. Kundu, R. Banerjee, *Adv. Mater.* **2024**, *36*, 2310938.

[4] C. Lin, J.-L. Li, X. Li, S. Yang, W. Luo, Y. Zhang, S.-H. Kim, D.-H. Kim, S. S. Shinde, Y.-F. Li, Z.-P. Liu, Z. Jiang, J.-H. Lee, *Nat. Catal.* **2021**, 4, 1012.

[5] C. Yang, Z.-D. Yang, H. Dong, N. Sun, Y. Lu, F.-M. Zhang, G. Zhang, *ACS Energy Lett.* **2019**, 4, 2251.

[6] S. Mondal, B. Mohanty, M. Nurhuda, S. Dalapati, R. Jana, M. Addicoat, A. Datta, B. K. Jena, A. Bhaumik, *ACS Catal.* **2020**, 10, 5623.

[7] C. Liu, F. Liu, H. Li, J. Chen, J. Fei, Z. Yu, Z. Yuan, C. Wang, H. Zheng, Z. Liu, M. Xu, G. Henkelman, L. Wei, Y. Chen, *ACS Nano* **2021**, 15, 3309.

[8] R. Wang, Z. Zhang, J. Suo, L. Liao, L. Li, Z. Yu, H. Zhang, V. Valtchev, S. Qiu, Q. Fang, *Chem. Eng. J.* **2023**, 478, 147403.

[9] W. Li, J. Wang, C. Jia, J. Chen, Z. Wen, A. Huang, *J. Colloid Interface Sci.* **2023**, 650, 275.

[10] C. J. Weifeng Xia, Rui Wang, Shilun Qiu, Qianrong Fang, *Acta Phys. -Chim. Sin.* **2023**, 39, 2212057.

[11] Y.-X. Lin, W.-J. Feng, J.-J. Zhang, Z.-H. Xue, T.-J. Zhao, H. Su, S.-I. Hirano, X.-H. Li, J.-S. Chen, *Angew. Chem. Int. Ed.* **2018**, 57, 12563.

[12] A. Sadhukhan, A. Karmakar, K. Koner, S. Karak, R. K. Sharma, A. Roy, P. Sen, K. K. Dey, V. Mahalingam, B. Pathak, S. Kundu, R. Banerjee, *Adv. Mater.* **2024**, 36, 2310938.

[13] S. Karak, K. Koner, A. Karmakar, S. Mohata, Y. Nishiyama, N. T. Duong, N. Thomas, T. G. Ajithkumar, M. S. Hossain, S. Bandyopadhyay, S. Kundu, R. Banerjee, *Adv. Mater.* **2024**, 36, 2209919.

[14] Y. Ge, Z. Lyu, M. Marcos-Hernández, D. Villagrán, *Chem. Sci.* **2022**, 13, 8597.

[15] S. Gopi, K. Giribabu, M. Kathiresan, *ACS Omega* **2018**, 3, 6251.

[16] S. Halder, A. K. Pradhan, P. Sivasakthi, P. K. Samanta, C. Chakraborty, *Mater. Today Chem.* **2023**, 32, 101649.

[17] N. Gupta, S. Halder, R. P. Behere, P. Singh, S. Kanungo, M. Dixit, C. Chakraborty, B. K. Kuila, *ACS Appl. Mater. Interfaces* **2023**, 15, 29042.

[18] C. Mo, J. Jian, J. Li, Z. Fang, Z. Zhao, Z. Yuan, M. Yang, Y. Zhang, L. Dai, D. Yu, *Energy Environ. Sci.* **2018**, 11, 3334.

[19] T. Y. Ma, S. Dai, M. Jaroniec, S. Z. Qiao, *Angew. Chem. Int. Ed.* **2014**, 53, 7281.

[20] J. Tian, Q. Liu, A. M. Asiri, K. A. Alamry, X. Sun, *ChemSusChem* **2014**, 7, 2125.

[21] Z. Wen, S. Ci, Y. Hou, J. Chen, *Angew. Chem. Int. Ed.* **2014**, 53, 6496.

[22] C. Tang, H.-F. Wang, X. Chen, B.-Q. Li, T.-Z. Hou, B. Zhang, Q. Zhang, M.-M. Titirici, F. Wei, *Adv. Mater.* **2016**, 28, 6845.

[23] S. Chen, J. Duan, M. Jaroniec, S.-Z. Qiao, *Adv. Mater.* **2014**, 26, 2925.

[24] J. Zhao, Q. Li, Q. Zhang, R. Liu, *Chem. Eng. J.* **2022**, 431, 133730.

[25] H. Li, B. Ren, W. Liu, L. Jing, R. Y. Tay, S. H. Tsang, L. Ricardez–Sandoval, A. Yu, E. H. T. Teo, *Nano Energy* **2021**, 88, 106246.

[26] S. Lu, Y. Shi, W. Zhou, Z. Zhang, F. Wu, B. Zhang, *J. Am. Chem. Soc.* **2022**, 144, 3250.

[27] H. Lei, M. Cui, Y. Huang, *ACS Appl. Mater. Interfaces* **2022**, 14, 34793.

[28] G. Yasin, S. Ibrahim, S. Ibraheem, S. Ali, R. Iqbal, A. Kumar, M. Tabish, Y. Slimani, T. A. Nguyen, H. Xu, W. Zhao, *J. Mater. Chem. A* **2021**, 9, 18222.

[29] X. Zheng, Y. Qian, H. Gong, W. Shi, J. Yan, W. Wang, X. Guo, J. Zhang, X. Cao, R. Yang, *Appl. Catal. B: Environ.* **2022**, 319, 121937.

[30] H.-F. Wang, C. Tang, Q. Zhang, *Catal. Today* **2018**, 301, 25.

[31] T. V. Tam, S. G. Kang, M. H. Kim, S. G. Lee, S. H. Hur, J. S. Chung, W. M. Choi, *Adv. Energy Mater.* **2019**, 9, 1900945.

[32] G.-L. Chai, K. Qiu, M. Qiao, M.-M. Titirici, C. Shang, Z. Guo, *Energy Environ. Sci.* **2017**, 10, 1186.

[33] D. Lyu, S. Yao, Y. Bahari, S. W. Hasan, C. Pan, X. Zhang, F. Yu, Z. Q. Tian, P. K. Shen, *Appl. Mater. Today* **2020**, 20, 100737.

[34] Y. Yin, X. Sun, M. Zhou, X. Zhao, J. Qin, S.-Z. Qiao, X.-W. Du, J. Yang, *ChemCatChem* **2019**, 11, 6131.

[35] Z. Wu, Y. Yu, G. Zhang, Y. Zhang, R. Guo, L. Li, Y. Zhao, Z. Wang, Y. Shen, G. Shao, *Adv. Sci.* **2022**, 9, 2200614.

[36] M. Li, Z. Liu, F. Wang, J. Xuan, *J. Energy Chem.* **2017**, 26, 422.

[37] B. Fang, J. Yang, C. Chen, C. Zhang, D. Chang, H. Xu, C. Gao, *ChemCatChem* **2017**, 9, 4520.

[38] Z. Yuan, J. Li, M. Yang, Z. Fang, J. Jian, D. Yu, X. Chen, L. Dai, *J. Am. Chem. Soc.* **2019**, 141, 4972.

[39] B. Z. Desalegn, H. S. Jadhav, J. G. Seo, *ChemCatChem* **2019**, 11, 2870.
[40] Z. Xiao, X. Huang, L. Xu, D. Yan, J. Huo, S. Wang, *Chem. Commun.* **2016**, 52, 13008.
[41] M. Zhao, T. Li, L. Jia, H. Li, W. Yuan, C. M. Li, *ChemSusChem* **2019**, 12, 5041.
[42] Y. Zhang, J. Liu, Z. Fang, X. Lin, W. Zhang, D. Yu, *Chemistry – An Asian Journal* **2020**, 15, 1544.
[43] M. Zhao, J. Zhang, H. Xiao, T. Hu, J. Jia, H. Wu, *Chem. Commun.* **2019**, 55, 1635.
[44] J. Zhang, M. Ren, L. Wang, Y. Li, B. I. Yakobson, J. M. Tour, *Adv. Mater.* **2018**, 30, 1707319.
[45] Y. Chang, A. Nie, S. Yuan, B. Wang, C. Mu, J. Xiang, B. Yang, L. Li, F. Wen, Z. Liu, *Nanotechnology* **2019**, 30, 035701.
[46] R. Prasannachandran, T. V. Vineesh, A. Anil, B. M. Krishna, M. M. Shaijumon, *ACS Nano* **2018**, 12, 11511.
[47] J. Sun, S. E. Lowe, L. Zhang, Y. Wang, K. Pang, Y. Wang, Y. Zhong, P. Liu, K. Zhao, Z. Tang, H. Zhao, *Angew. Chem. Int. Ed.* **2018**, 57, 16511.
[48] Y. Zhang, X. Fan, J. Jian, D. Yu, Z. Zhang, L. Dai, *Energy Environ. Sci.* **2017**, 10, 2312.
[49] Y. Lin, K.-H. Wu, Q. Lu, Q. Gu, L. Zhang, B. Zhang, D. Su, M. Plodinec, R. Schlögl, S. Heumann, *J. Am. Chem. Soc.* **2018**, 140, 14717.
[50] X. Lu, W.-L. Yim, B. H. R. Suryanto, C. Zhao, *J. Am. Chem. Soc.* **2015**, 137, 2901.
[51] J. Zhang, L. Dai, *Angew. Chem. Int. Ed.* **2016**, 55, 13296.
[52] H. B. Yang, J. Miao, S.-F. Hung, J. Chen, H. B. Tao, X. Wang, L. Zhang, R. Chen, J. Gao, H. M. Chen, L. Dai, B. Liu, *Sci. Adv.* **2016**, 2, e1501122.
[53] C. Hu, L. Dai, *Adv. Mater.* **2017**, 29, 1604942.
[54] A. M. El-Sawy, I. M. Mosa, D. Su, C. J. Guild, S. Khalid, R. Joesten, J. F. Rusling, S. L. Suib, *Adv. Energy Mater.* **2016**, 6, 1501966.
[55] T. Sun, J. Wang, C. Qiu, X. Ling, B. Tian, W. Chen, C. Su, *Adv. Sci.* **2018**, 5, 1800036.
[56] Q. Wang, Y. Ji, Y. Lei, Y. Wang, Y. Wang, Y. Li, S. Wang, *ACS Energy Lett.* **2018**, 3, 1183.
[57] A. Muthurasu, I. Pathak, D. Acharya, Y. R. Rosyara, H. Y. Kim, *J. Mater. Chem. A* **2024**, 12, 1826.
[58] F. Qiang, J. Feng, H. Wang, J. Yu, J. Shi, M. Huang, Z. Shi, S. Liu, P. Li, L. Dong, *ACS Catal.* **2022**, 12, 4002.
[59] Z. Pei, H. Tan, J. Gu, L. Lu, X. Zeng, T. Zhang, C. Wang, L. Ding, P. J. Cullen, Z. Chen, S. Zhao, *Nat. Commun.* **2023**, 14, 818.
[60] C. Xing, Y. Xue, B. Huang, H. Yu, L. Hui, Y. Fang, Y. Liu, Y. Zhao, Z. Li, Y. Li, *Angew. Chem. Int. Ed.* **2019**, 58, 13897.
[61] Q. Hu, G. Li, X. Liu, B. Zhu, X. Chai, Q. Zhang, J. Liu, C. He, *Angew. Chem. Int. Ed.* **2019**, 58, 4318.
[62] S. S. Shinde, J.-Y. Yu, J.-W. Song, Y.-H. Nam, D.-H. Kim, J.-H. Lee, *Nanoscale Horiz.* **2017**, 2, 333.
[63] K. Kordek, L. Jiang, K. Fan, Z. Zhu, L. Xu, M. Al-Mamun, Y. Dou, S. Chen, P. Liu, H. Yin, P. Rutkowski, H. Zhao, *Adv. Energy Mater.* **2019**, 9, 1802936.
[64] M.-S. Balogun, W. Qiu, H. Yang, W. Fan, Y. Huang, P. Fang, G. Li, H. Ji, Y. Tong, *Energy Environ. Sci.* **2016**, 9, 3411.
[65] Z. Liu, Z. Zhao, Y. Wang, S. Dou, D. Yan, D. Liu, Z. Xia, S. Wang, *Adv. Mater.* **2017**, 29, 1606207.
[66] D. Li, B. Ren, Q. Jin, H. Cui, C. Wang, *J. Mater. Chem. A* **2018**, 6, 2176.
[67] X.-Z. Fan, Q.-Q. Pang, S.-S. Yi, X. Du, S. Zhang, Z.-Y. Liu, X.-Z. Yue, *Appl. Catal. B: Environ.* **2021**, 292, 120152.
[68] T. Y. Ma, J. Ran, S. Dai, M. Jaroniec, S. Z. Qiao, *Angew. Chem. Int. Ed.* **2015**, 54, 4646.
[69] N. Cheng, Q. Liu, J. Tian, Y. Xue, A. M. Asiri, H. Jiang, Y. He, X. Sun, *Chem. Commun.* **2015**, 51, 1616.
[70] S. Chen, J. Duan, J. Ran, S.-Z. Qiao, *Adv. Sci.* **2015**, 2, 1400015.
[71] X. Yu, M. Zhang, J. Chen, Y. Li, G. Shi, *Adv. Energy Mater.* **2016**, 6, 1501492.
[72] J. Guan, Z. Duan, F. Zhang, S. D. Kelly, R. Si, M. Dupuis, Q. Huang, J. Q. Chen, C. Tang, C. Li, *Nat. Catal.* **2018**, 1, 870.
[73] H. Fei, J. Dong, Y. Feng, C. S. Allen, C. Wan, B. Volosskiy, M. Li, Z. Zhao, Y. Wang, H. Sun, P. An, W. Chen, Z. Guo, C. Lee, D. Chen, I. Shakir, M. Liu, T. Hu, Y. Li, A. I. Kirkland, X. Duan, Y. Huang, *Nat. Catal.* **2018**, 1, 63.
[74] P. Kumar, K. Kannimuthu, A. S. Zeraati, S. Roy, X. Wang, X. Wang, S. Samanta, K. A. Miller, M. Molina, D. Trivedi, J. Abed, M. A. Campos Mata, H. Al-Mahayni, J. Baltrusaitis, G. Shimizu, Y. A. Wu, A. Seifitokaldani, E. H. Sargent, P. M. Ajayan, J. Hu, M. G. Kibria, *J. Am. Chem. Soc.* **2023**, 145, 8052.
[75] L. Bai, C.-S. Hsu, D. T. L. Alexander, H. M. Chen, X. Hu, *J. Am. Chem. Soc.* **2019**, 141, 14190.
[76] Y. Pan, K. Sun, S. Liu, X. Cao, K. Wu, W.-C. Cheong, Z. Chen, Y. Wang, Y. Li, Y. Liu, D. Wang, Q. Peng, C. Chen, Y. Li, *J. Am. Chem. Soc.* **2018**, 140, 2610.
[77] Y. Zheng, Y. Jiao, Y. Zhu, Q. Cai, A. Vasileff, L. H. Li, Y. Han, Y. Chen, S.-Z. Qiao, *J. Am. Chem. Soc.* **2017**, 139, 3336.
[78] M. Yu, C. Weidenthaler, Y. Wang, E. Budiyanto, E. Onur Sahin, M. Chen, S. DeBeer, O. Rüdiger, H. Tüysüz, *Angew. Chem. Int. Ed.* **2022**, 61, e202211543.
[79] P. He, X.-Y. Yu, X. W. Lou, *Angew. Chem. Int. Ed.* **2017**, 56, 3897.

[80] M.-Y. Ye, S. Li, X. Zhao, N. V. Tarakina, C. Teutloff, W. Y. Chow, R. Bittl, A. Thomas, *Adv. Mater.* **2020**, 32, 1903942.
[81] S. Wan, J. Qi, W. Zhang, W. Wang, S. Zhang, K. Liu, H. Zheng, J. Sun, S. Wang, R. Cao, *Adv. Mater.* **2017**, 29, 1700286.
[82] S. Chen, Z. Kang, X. Hu, X. Zhang, H. Wang, J. Xie, X. Zheng, W. Yan, B. Pan, Y. Xie, *Adv. Mater.* **2017**, 29, 1701687.
[83] S. Wahl, S. M. El-Refaei, A. G. Buzanich, P. Amsalem, K.-S. Lee, N. Koch, M.-L. Doublet, N. Pinna, *Adv. Energy Mater.* **2019**, 9, 1900328.
[84] Z.-H. Xue, H. Su, Q.-Y. Yu, B. Zhang, H.-H. Wang, X.-H. Li, J.-S. Chen, *Adv. Energy Mater.* **2017**, 7, 1602355.
[85] J. Masa, I. Sinev, H. Mistry, E. Ventosa, M. de la Mata, J. Arbiol, M. Muhler, B. Roldan Cuenya, W. Schuhmann, *Adv. Energy Mater.* **2017**, 7, 1700381.
[86] T. X. Nguyen, Y.-C. Liao, C.-C. Lin, Y.-H. Su, J.-M. Ting, *Adv. Funct. Mater.* **2021**, 31, 2101632.
[87] Z. Li, Z. Wang, S. Xi, X. Zhao, T. Sun, J. Li, W. Yu, H. Xu, T. S. Herng, X. Hai, P. Lyu, M. Zhao, S. J. Pennycook, J. Ding, H. Xiao, J. Lu, *ACS Nano* **2021**, 15, 7105.
[88] G. Fang, Q. Wang, J. Zhou, Y. Lei, Z. Chen, Z. Wang, A. Pan, S. Liang, *ACS Nano* **2019**, 13, 5635.
[89] K. Fan, H. Zou, Y. Lu, H. Chen, F. Li, J. Liu, L. Sun, L. Tong, M. F. Toney, M. Sui, J. Yu, *ACS Nano* **2018**, 12, 12369.
[90] Z. Zhang, C. Feng, X. Li, C. Liu, D. Wang, R. Si, J. Yang, S. Zhou, J. Zeng, *Nano Lett.* **2021**, 21, 4795.
[91] L. Bai, Z. Duan, X. Wen, R. Si, Q. Zhang, J. Guan, *ACS Catal.* **2019**, 9, 9897.
[92] X. Sun, S. Sun, S. Gu, Z. Liang, J. Zhang, Y. Yang, Z. Deng, P. Wei, J. Peng, Y. Xu, C. Fang, Q. Li, J. Han, Z. Jiang, Y. Huang, *Nano Energy* **2019**, 61, 245.
[93] C. Lin, Y. Zhao, H. Zhang, S. Xie, Y.-F. Li, X. Li, Z. Jiang, Z.-P. Liu, *Chem. Sci.* **2018**, 9, 6803.
[94] Y. Cheng, H. Guo, X. Li, X. Wu, X. Xu, L. Zheng, R. Song, *Chem. Eng. J.* **2021**, 410, 128359.
[95] Y. Xu, W. Zhang, Y. Li, P. Lu, Z.-S. Wu, *J. Energy Chem.* **2020**, 43, 52.
